# RIPK3 activation induces TRIM28 derepression in cancer cells and enhances the anti-tumor microenvironment

**DOI:** 10.1186/s12943-021-01399-3

**Published:** 2021-08-21

**Authors:** Han-Hee Park, Hwa-Ryeon Kim, Sang-Yeong Park, Sung-Min Hwang, Sun Mi Hong, Sangwook Park, Ho Chul Kang, Michael J. Morgan, Jong-Ho Cha, Dakeun Lee, Jae-Seok Roe, You-Sun Kim

**Affiliations:** 1 Department of Biochemistry, Ajou University School of Medicine, Suwon, 16499 South Korea; 2 Department of Biomedical Sciences, Graduate School, Ajou University, Suwon, 16499 South Korea; 3 Department of Biochemistry, College of Life Science and Biotechnology, Yonsei University, Seoul, 03722 South Korea; 4 Department of Physiology, Ajou University School of Medicine, Suwon, 16499 South Korea; 5 Department of Natural Sciences, Northeastern State University, Tahlequah, OK 74464 USA; 6 Department of Biomedical Sciences, College of Medicine, Inha University, Incheon, 22212 South Korea; 7 Department of Biomedical Science and Engineering, Graduate School, Inha University, Incheon, 22212 South Korea; 8 Department of Pathology, Ajou University School of Medicine, Suwon, 16499 South Korea

**Keywords:** RIPK3, TRIM28, NF-κB, Transcriptional regulator, Chromatin, Immunostimulatory cytokines

## Abstract

**Background:**

Necroptosis is emerging as a new target for cancer immunotherapy as it is now recognized as a form of cell death that increases tumor immunogenicity, which would be especially helpful in treating immune-desert tumors. De novo synthesis of inflammatory proteins during necroptosis appears especially important in facilitating increased anti-tumor immune responses. While late-stage transcription mediated by NF-κB during cell death is believed to play a role in this process, it is otherwise unclear what cell signaling events initiate this transactivation of inflammatory genes.

**Methods:**

We employed tandem-affinity purification linked to mass spectrometry (TAP-MS), in combination with the analysis of RNA-sequencing (RNA-Seq) datasets to identify the Tripartite Motif Protein 28 (TRIM28) as a candidate co-repressor. Comprehensive biochemical and molecular biology techniques were used to characterize the role of TRIM28 in RIPK3 activation-induced transcriptional and immunomodulatory events. The cell composition estimation module was used to evaluate the correlation between RIPK3/TRIM28 levels and CD8^+^ T cells or dendritic cells (DC) in all TCGA tumors.

**Results:**

We identified TRIM28 as a co-repressor that regulates transcriptional activity during necroptosis. Activated RIPK3 phosphorylates TRIM28 on serine 473, inhibiting its chromatin binding activity, thereby contributing to the transactivation of NF-κB and other transcription factors, such as SOX9. This leads to elevated cytokine expression, which then potentiates immunoregulatory processes, such as DC maturation. The expression of RIPK3 has a significant positive association with the tumor-infiltrating immune cells populations in various tumor type, thereby activating anti-cancer responses.

**Conclusion:**

Our data suggest that RIPK3 activation-dependent derepression of TRIM28 in cancer cells leads to increased immunostimulatory cytokine production in the tumor microenvironment, which then contributes to robust cytotoxic anti-tumor immunity.

**Supplementary Information:**

The online version contains supplementary material available at 10.1186/s12943-021-01399-3.

## Background

Cell death in mammalian cells occurs via multiple mechanisms (e.g., apoptosis, necrosis, pyroptosis, etc.) in response to different stresses; the abnormal regulation of cell death contributes to various human diseases such as neurodegeneration, autoimmune diseases, infectious diseases, and cancer [1, 2]. Necroptosis is a form of regulated necrotic cell death; its essential molecular machinery consist of two receptor-interacting protein kinases (RIPK1 and RIPK3), and mixed lineage kinase domain-like pseudokinase (MLKL) [3]. Phosphorylation of RIPK3 is an essential aspect of core necroptotic pathway, and this subsequently leads to phosphorylated MLKL, which induces oligomerization and translocation to the plasma membrane where it causes membrane permeabilization [4, 5]. Necroptotic cells may play multiple roles in innate immunity and shape the subsequent adaptive immunity through the release of endogenous danger signals known as damage-associated molecular patterns (DAMPs) [6, 7]. Mounting evidence suggests that following the induction of necroptosis, the de novo synthesis of cytokines and chemokines occurs as cells are dying, which then affect immune processes [8–10]. Indeed, the activation of RIPK1/RIPK3 leads to the upregulation of inflammatory chemokines that promote the cross-priming of CD8^+^ T cell vaccination responses [11–13]; the presence of intratumoral chemokines is positively correlated with cytotoxic CD8^+^ T cell (CTL) infiltration [14] indicating that the activation of necroptosis signaling provides anti-tumor immunity.

Some studies have recently suggested mechanisms for the production of immunostimulatory cytokines during necroptosis [9, 11, 12]. These reports revealed an NF-κB transcriptional and translational activity that is engaged during RIPK1/RIPK3 activation-dependent necroptosis. Certainly, tumor necrosis factor (TNF)-mediated necroptosis enhances inflammatory cytokine gene transcription through sustained NF-κB activation in large part through well-known mechanisms; however, how RIPK3 activation itself leads to sustained NF-κB activation is not fully understood. Other mechanisms underlying the cell-intrinsic activation of cytokine production may also exist [9].

TRIM28, one of the 60 TRIM family proteins, is a transcriptional regulator involved in gene expression, mediated in part through its interaction with Krüppel-associated box repression domains often found in transcription factors [15, 16]. TRIM28 is located in the heterochromatin in conjunction with heterochromatin protein 1 (HP1), where the plant homeodomain finger and bromodomain, located in the carboxyl-terminus of TRIM28, recruits various transcriptional co-repressors, including the nucleosome remodeling deacetylase (NuRD), histone deacetylase complex, and histone H3 lysine 9-specific methyltransferase SETDB1 to repress gene expression [17, 18]. The co-repressor function of TRIM28 have been shown recently to be linked to the development of various cancers, such as non-small cell lung cancer, breast cancer, cervical cancer, colon cancer, gastric cancer, and ovarian cancer [19–24]. However, how TRIM28 is involved in active transcription triggered by external stimuli remains elusive.

Here, we show that TRIM28 is a negative transcriptional regulator that is itself negatively regulated when cells undergoing necroptosis continue de novo synthesis of immunostimulatory cytokines. TRIM28 antagonizes NF-κB transactivation independent of p65 chromatin occupancy, but RIPK3 activation-mediated phosphorylation of TRIM28 at serine 473 facilitates its derepression. Moreover, RIPK3 activation triggers a remarkable reduction in TRIM28 binding events in chromatin that leads to increased SOX9 transcription factor activity. Our results reveal a new necroptosis-mediated transcription circuit that is modulated by RIPK3 activation-dependent de-repression of TRIM28, which provides a mechanism to promote robust anti-tumor immunity and contributes to tumor immunogenicity.

## Methods

### Aim and design

The aim of this study was to identify factors, including proteins and cell signaling events that influence the immunogenicity of necroptotic cell death. Previous reports have shown that transcription and protein synthesis continues to occur after the necroptotic cell death process has begun, perhaps even after cell membrane integrity has been lost, and that proteins produced during this stage affect how the immune cell responds to the dying cell. We therefore sought to use mass spectrometry (TAP-MS), in combination with the analysis of RNA-sequencing (RNA-Seq) datasets to identify such late-stage protein factors. As RIPK1/RIPK3 is consistently identified as a key essential player for the immunogenicity of dying cells, this protein was used as bait for the TAP-MS. The remainder of the study was designed to characterize the function of the identified protein using standard biochemical and molecular biology techniques.

## Experimental models and subject details

### Cell lines and culture conditions

MEF, 293 T, 293A, NIH-3T3, Raw 264.7, L929, HT-29, and HeLa cells were grown in Dulbecco’s modified Eagle’s medium (DMEM) supplemented with 10% fetal bovine serum (FBS). ML-1 and SNU620 cells were maintained in Roswell Park Memorial Institute (RPMI) 1640 medium supplemented with 10% FBS. To generate cell lines stably expressing the RIPK3 construct, HeLa cells were infected with the pLX303-hRIPK3 lentivirus. To generate the RIPK3-knockdown model, HT-29, ML-1, and SNU620 cells were infected with the RIPK3 shRNA lentivirus. To generate TRIM28-knockdown cells, HT-29, HeLa (RIPK3), SNU620, and L929 cells were infected with the TRIM28 shRNA lentivirus. To generate cell lines stably expressing the TRIM28 S473 wild type or mutant constructs, HT-29 (shTRIM28) cells were infected with the pLX303-TRIM28 S473 wild type or mutants lentivirus. All cells were cultured in 37 °C, 5% CO_2_ incubators. All the cell lines regularly tested for mycoplasma contamination.

## Method details

### Lentiviral shRNA experiments

MISSION® shRNA plasmids targeting hRIPK3 mRNA (NM_006871), hTRIM28 mRNA (NM_005762), mTRIM28 mRNA (NM_011588), and the non-targeting control (NM_027088) were purchased from Sigma-Aldrich. shRNA plasmids targeting hTRIM28 mRNA were tested with 5 different clone (#1: 199141, #2: 358545, #3: 18001, #4: 18002, #5: 17998) and most of data were represented with #5 clone. Lentiviral plasmids were transfected into 293 T cells using Lipofectamine 2000 (Invitrogen) for 48 h. Then, pseudoviral particles were collected, and infected to cells in the presence of polybrene (8 μg/mL). The cells were selected using puromycin 2 days after infection.

### Primary culture and activation of BMDCs

Bone marrow (BM) cells were cultured in RPMI medium containing 20 ng/ml GM-CSF for 5 days to generate immature DCs (iDCs). To activate iDCs, cells were exposed to the conditioned medium from necroptotic cells for 16 h. Cell surface markers were analyzed using flow cytometry (Canto II flow cytometer, BD), and data were analyzed using FlowJo™ software (BD).

### Antibodies and chemical reagents

Antibodies used for immunoblot and immunoprecipitation analysis were as follows: anti-GFP (Santa Cruz, 9996, 1:1000), anti-Myc (Cell Signaling Technology, 2272, 1:1000), anti-HA (Cell Signaling Technology, 3724, 1:1000), anti-Flag (Sigma Aldrich, F3165, 1:1000), anti-GAPDH (Santa Cruz, 25,778, 1:2500), anti-TOPOIIα (Santa Cruz, 13,058, 1:2000), anti-HSP90 (Cell Signaling Technology, 4874, 1:1000), anti-LAMIN A/C (Santa Cruz, 7293, 1:1000), anti-SP1 (Santa Cruz, 17,824, 1:1000), anti-ACTIN (Santa Cruz, 47,778, 1:5000), anti-VINCULIN (Sigma Aldrich, V9131, 1:5000), anti-PARP (Cell Signaling Technology, 9542, 1:1000), anti-RIPK1 (BD, 610458, 1:1000), anti-p-RIPK1 (Cell Signaling Technology, 65,746, 1:1000), anti-p-RIPK1 (Cell Signaling Technology, 31,122, 1:1000), anti-RIPK3 (Cell Signaling Technology, 13,526, 1:1000), anti-p-RIPK3 (S227) (Abcam, ab209384, 1:1000), anti-p-MLKL (Abcam, ab187091, 1:1000), anti-p-MLKL (Abcam, ab196436, 1:5000), anti- p105/p50 (Cell Signaling Technology, 13,586, 1:1000), anti-p65 (Cell Signaling Technology, 6956, 1:1000), anti-p-p65 (Cell Signaling Technology, 3033, 1:1000), anti-IκBα (Santa Cruz, 371, 1:1000), anti-p-IκBα (Cell Signaling Technology, 2859, 1:1000), anti-TRIM28 (Cell Signaling Technology, 4123, 1:1000), anti-p-TRIM28 (S473) (BioLegend, 654,102, 1:2000), anti-p-TRIM28 (S824) (Cell Signaling Technology, 4127, 1:1000), anti-IL-8 (Proteintech, 17,038–1-AP, 1:1000), anti-p-ATM (ECM Biosciences, AM3661, 1:1000), and anti-γ-H2AX (Cell Signaling Technology, 9718, 1:1000). TNF-α and zVAD were purchased from R&D Systems. The SMAC mimetic (LCL-161) was obtained from Adooq Bioscience. Necrostatin-1, doxorubicin, and etoposide were from Sigma-Aldrich. NSA and GSK’872 were purchased from Merck. Cycloheximide was from Calbiochem. Dabrafenib was obtained from Selleck Chemicals. GST-TRAIL was purified in the laboratory.

### Plasmid construction and transfection

Flag-RIPK3, GFP-TRIM28, Myc-TRIM28, HA-TRIM28, and Flag-TRIM28 were generated using LR cloning (Invitrogen, LR clonase). Various TRIM28 and RIPK3 mutants were generated using a site-directed mutagenesis kit (iNtRON Biotechnology, Muta-Direct). For transient expression, constructs were transfected into cells using polyethylenimine (Polysciences) or Lipofectamine Plus (Invitrogen).

### Cytotoxicity assays

Cell viability was determined using the tetrazolium dye colorimetric test (MTT assay) (Sigma Aldrich, M5655), and absorbance at 570 nm was measured. Lactate dehydrogenase (LDH) leakage was quantified using a CytoTox 96® Non-Radioactive Cytotoxicity Assay kit (Promega, G1780) according to the manufacturer’s instructions. LDH absorbance was measured at 490 nm. Absorbance signal was measured using a POLARstar OPTIMA Multidetection Microplate Reader. the mean ± STDEV of duplicates is presented.

### Immunoprecipitation and immunoblot analysis

For immunoprecipitation, cells were lysed in M2 buffer (20 mM Tris at pH 7, 0.5% NP-40, 250 mM NaCl, 3 mM EDTA, 3 mM EGTA, 2 mM DTT, 0.5 mM PMSF, 20 mM β-glycerol phosphate, 1 mM sodium vanadate, and 1 mg/ml leupeptin). Equal amounts of cell lysates were mixed and precipitated with antibodies and protein A-sepharose or protein G-agarose beads overnight or 3 h at 4 °C. Bound proteins were removed by boiling in SDS, resolved by SDS-PAGE and immunoblotting, and visualized by enhanced chemiluminescence (Pierce™ ECL Western Blotting Substrate, 32,106).

### Cell fractionation assay

Nuclear and cytoplasmic extractions were performed using a NE-PER nuclear and cytoplasmic extraction reagent (Thermo, #78833) according to the manufacturer’s instructions. Equal amounts of protein were loaded onto SDS-PAGE gel. TopoIIα, Lamin A/C, and Sp1 were used for nuclear fraction normalization, whereas Hsp90 and GAPDH were used for cytosolic fraction normalization.

### TAP & MASS analysis

The human gene of RIPK3 wild type (RIPK3 WT) and RIPK3 kinase-dead (RIPK3 KD) were subcloned in frame with a TAP-tag plasmid and were transiently transfected into the 293 T cells. Following 18 h of expression, the cells were lysed and subjected to two steps (streptavidin and calmodulin) of purification according to the optimized TAP protocol. A representative result of two purification steps of TAP-mock, TAP-RIPK3 WT, or TAP-RIPK3-KD was determined by immunoblot using anti-RIPK3 and anti-phospho-RIPK3 antibodies. To visualize the TAP-RIPK3 binding proteins from purified samples, each sample was loaded into 8–16% SDS-PAGE and stained with Coomassie brilliant blue. All protein bands were excised from the gel and subjected to LC–MS/MS for molecular identities.

### TCGA analysis

TCGA analysis was performed with TIMER2.0 based on R package which integrates six state-of-the-art algorithms, including TIMER, xCell, MCP-counter, CIBERSORT, EPIC, and quanTIseq [25]. Cellular composition estimation module was used to generate a heatmap table of the Spearman’s correlations between the expression of IFN-β/RIPK3/TRIM28 and all types of CD8^+^ T cell or DC across all TCGA tumors. The Gene_DE module was used to compare TRIM28 level between tumor and matched normal tissues across all TCGA tumors.

### ChIP-Seq library construction and analysis

Cells were dissociated into single cells to yield at least 2 × 10^7^ cells, crosslinked for 10 min with formaldehyde (1% final), and quenched with 0.125 M glycine for 10 min. PBS-washed cells were subjected to ChIP assay as previously described [26]. ChIP-seq libraries were constructed using a NEXTflex™ ChIP-seq kit (Cat# NOVA-5143-02; PerkinElmer) according to the manufacturer’s instructions. Sequencing was peformed on the NextSeq platform to obtain single-end reads of 50 bases. Raw reads were mapped to the reference mouse genome assembly (mm10) using Bowtie2 and SAMtools. The makeBigWig tool of the HOMER suite was used to generate browser tracks for visualization using the UCSC genome browser. The GEO accession numbers for the raw and processed ChIP-Seq data reported in this article are GSE 178847.

### Immunofluorescence analysis

Cells were washed twice with DPBS, fixed in 4% paraformaldehyde for 10 min, and permeabilized with 0.25% Triton X-100 for 15 min. After incubation in a blocking buffer for 30 min, the cells were incubated overnight at 4 °C with the following primary antibodies: anti-p-TRIM28 (S473) (BioLegend, 654,102), anti-p-TRIM28 (S824) (Cell Signaling Technology, 4127), anti-TRIM28 (Cell Signaling Technology, 4123), and anti-p-MLKL (Abcam, ab187091). Then, they were incubated with the following Alexa Fluor secondary antibodies (Invitrogen) for 1 h at room temperature: 594-conjugated mouse (A21125), 488-conjugated mouse (A11001), and 488-conjugated rabbit (A11008). A mounting medium containing DAPI (Vector Laboratories, 94,010) was used for counterstaining.

### Flow cytometry

Cell suspensions were stained on ice for 20 min in the dark with various combinations of the following fluorochrome-conjugated antibodies: CD11c (BioLegend, 117,318), F4/80 (BioLegend, 123,128), CD86 (BioLegend, 105,012), and MHC II (Biolegend, 107,606). Zombie (BioLegend, 77,184) and FITC Annexin V Apoptosis Detection kit (BD Biosciences, 556,547) were used according to the manufacturer’s instructions.

### Real-time PCR

RNA was extracted using the TRIzol reagent (Life Technologies, 15,596,018). Total RNA (1 μg) from each sample was used for cDNA synthesis using MMLV reverse transcriptase (MGmed, MR10601). Equal amounts of cDNA product were used in real-time PCR with GoTaq® qPCR Master Mix (Promega, A6001). Gene expression was normalized to that of actin. Real-time PCR was performed on CFX Connect™. The oligonucleotides are listed in the Table 1.

**Table 1:** Primer sequences used in PCR

Gene name	Specis		Sequence
*ACTIN*	human	F	GGACTTCGAGCAAGAGATGG
*ACTIN*	human	R	AGCACTGTGTTGGCGTACAG
*CXCL1*	human	F	AGGGAATTCACCCCAAGAAC
*CXCL1*	human	R	TGGATTTGTCACTGTTCAGCA
*IL-8*	human	F	TCTGCAGCTCTGTGTGAAGG
*IL-8*	human	R	AATTTCTGTGTTGGCGCAGT
*IL-1β*	human	F	AAGTACCTGAGCTCGCCAGTGA
*IL-1β*	human	R	TGCTGTAGTGGTGGTCGGAGAT
*CCL4*	human	F	AAGCTCTGCGTGACTGTCCT
*CCL4*	human	R	GCTTGCTTCTTTTGGTTTGG
*TNF-α*	human	F	CAGAGGGCCTGTACCTCATC
*TNF-α*	human	R	GGAAGACCCCTCCCAGATAG
*TRIM28*	human	F	CTCGGGATGGTGAACGTACT
*TRIM28*	human	R	GCAATGTTGCATGTTTGTCC
*Il-6*	mouse	F	AGTTCCTCTCTGCAAGAGACT
*Il-6*	mouse	R	ATGTGTAATTAAGCCTCCGACTT
*Ccl4*	mouse	F	GCCCTCTCTCTCCTCTTGCT
*Ccl4*	mouse	R	GTCTGCCTCTTTTGGTCAGG
*Cd80*	mouse	F	CCATGTCCAAGGCTCATTCT
*Cd80*	mouse	R	TTCCCAGCAATGACAGACAG
*Cd40*	mouse	F	CCTGGCTTTGGAGTTATGGA
*Cd40*	mouse	R	CCGGGACTTTAAACCACAGA
*Cd86*	mouse	F	ATGCACCATGGGCTTGGCAA
*Cd86*	mouse	R	AACTTTTGCTGGTCCTGCCAAA
*Actin*	mouse	F	CCACACCTTCTACAATGAGC
*Actin*	mouse	R	TGAGGTAGTCAGTCAGGTC

### NF-κB reporter assay

Cells were transfected with NF-κB Luc plasmid (firefly luciferase) or the control plasmid (Renilla luciferase) using PEI for 24 h and treated with TNF-α or TSZ for 6 h. Luciferase activity was measured using the Dual-Luciferase Reporter Assay System (Promega, E1910).

#### Cytokine array

Cytokines from medium conditioned either by HT-29 cells expressing TRIM28 shRNA (shTRIM28) or a non-silencing control (shNC) were treated with TSZ for 6 h were analyzed with human cytokine antibody array (ab169817; Abcam) according to the manufacturer’s instructions. Signals were detected with chemiluminescence reaction.

#### Human gastric tumor tissue preparation

We collected formalin-fixed, paraffin-embedded (FFPE) tissues from 338 patients with gastric cancer who underwent surgery between January 2005 and December 2006 at the Ajou University Hospital, Republic of Korea. Clinical data were retrieved from patient medical records. Patients were excluded if they had been treated with preoperative chemotherapy or radiotherapy. Patients with distant metastasis at the time of surgery were also excluded.

#### Immunohistochemistry

The immunohistochemistry was carried out on 4-μm-thick, FFPE tissue sections using an automated immunostainer (Ventana Medical Systems Inc., Tucson, AZ) according to the manufacturer’s instructions. The primary antibodies used were as follows: anti-RIPK3, 1:250 (Thermo Fisher, Rockford, IL); anti-CD8, predilution (Roche, Tucson, AZ); and anti-Granzyme B, 1:50 (Cell Marque, Rocklin, CA). The expression of RIPK3 was semi-quantitatively evaluated as follows: staining intensity was graded as 0 (absent), 1 (weak), or 2 (strong), while staining area was graded as 1 (0–25%), 2 (26–50%), 3 (51–75%), or 4 (76–100%). Then, total immunostaining score was determined by multiplying staining intensity and area (range, 0–8). We considered cases with an immunostaining score greater than or equal to 4 as RIPK3-high.

#### Quantification and statistical analysis

Each experiment was repeated three times or more. Statistical analysis was performed using unpaired Student’s *t*-test in Graphpad prism 9. Data are presented as means ±SEM. ****p < 0.0001; ***p < 0.001; **p < 0.01; *p < 0.05. ns, not significant.

## Results

### NF-κB transactivation alone is insufficient to explain necroptosis-specific cytokine production

**Fig. 1:**
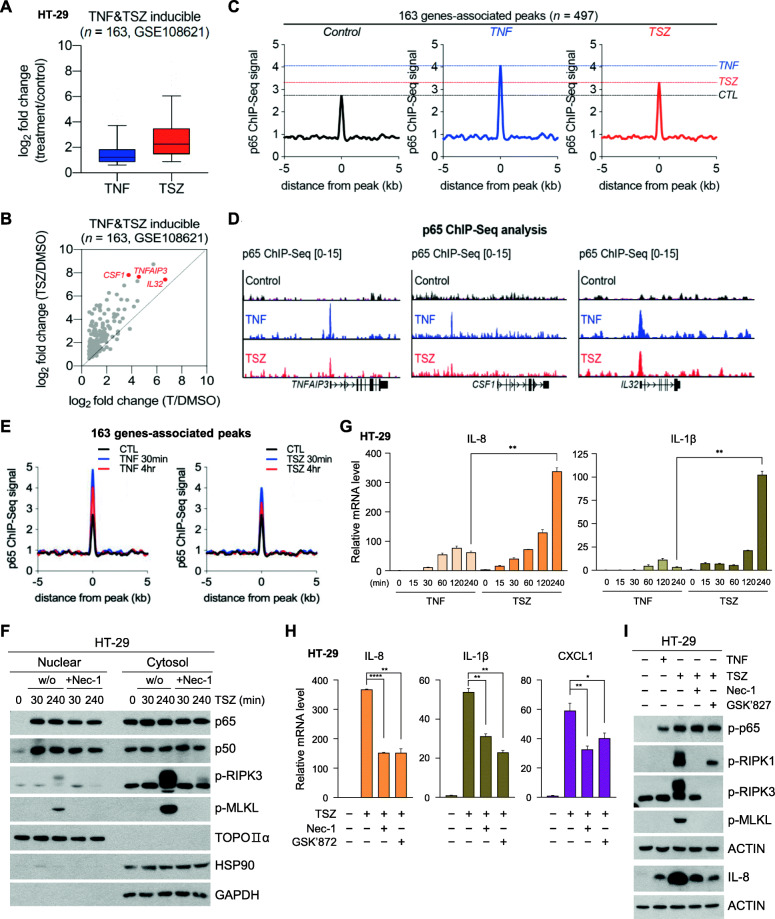
Necroptosis-dependent transcriptional hyperactivation. **(A-I)** All experiments were used with HT-29 colon cancer cells which is well established for necroptosis pathway. **(A)** RNA-seq-based expression of 163 genes in HT-29 cells treated with TNF-α or TNF-α + SMAC + zVAD (herein, TSZ). **(B)** Scatter plot comparing the expression of TNF-α- and TSZ-inducible genes (log2 scale) in the TNF-α /DMSO RNA-seq (X-axis) versus the TSZ/DMSO RNA-seq (Y-axis). HT-29 cells were treated with TNF-α or TSZ. **(C)** Metagene representation of the ChIP-seq signal for p65 across 497 regions and 163 gene-associated peaks in HT-29 cells treated with TNF-α (30 ng/ml) or TSZ (TNF-α (30 ng/ml) + SMAC (200 nM) + zVAD (20 μM), hereafter referred to as TSZ for 4 h. Metagenes centered on the middle of 497 regions and 10 Kb around the center of 497 regions are displayed. **(D)** ChIP-seq profiles of p65 in HT-29 cells treated with TNF-α or TSZ for 4 h at the *TNFAIP3*, *CSF1*, and *IL32* loci. **(E)** Metagene representation of the ChIP-seq signal for p65 across 497 regions in HT-29 cells treated with TNF-α or TSZ for 30 min or 4 h. Metagenes centered on the middle of 497 regions and 10 Kb around the center of 497 regions are displayed. **(F)** Western blot analysis of the nuclear and cytosol fractions. HT-29 cells were pretreated with necrostatin-1 (Nec-1, 40 μM) for 1 h and with TSZ for 30 min and 4 h. **(G)** HT-29 cells were treated with TSZ for the indicated periods, and *IL-8* and *IL-1β* mRNA levels were measured by qPCR. **(H)** HT-29 cells were pretreated with Nec-1 and GSK’872 (10 μM) for 1 h and with TSZ for 4 h. *IL-8*, *IL-1β*, and *CXCL1* mRNA levels were measured by qPCR. **(I)** HT-29 cells were pretreated with Nec-1 and GSK’872 for 1 h and with TSZ or TNF for 4 h. Cell lysates were analyzed by western blotting

It has been reported that the RIPK1/RIPK3 necrosome complex activation triggers cytokine gene transcription through a cell-autonomous mechanism involving NF-κB; this is proposed to occur independently of damage-associated molecular patterns (DAMP) release by the dying cells [9]. While NF-κB is reported to be critical for cytokine transcription in this case, it remains unknown if NF-κB transactivation is sufficient to trigger necroptosis-mediated transcriptional activation on its own. Moreover, while RIPK1 activates NF-κB through well-known mechanism, this mechanism does not require RIPK3, so it is unclear as to how the RIPK3-dependent necrosome influences NF-κB activity. To explore the NF-κB functional dependency of necrosome activation, we analyzed a publicly available RNA-sequencing (RNA-Seq) dataset acquired from a previous study [9]. We identified 163 genes whose expression was increased by more than two-fold upon stimulation with TNF-α alone or simultaneous stimulation with TNF-α (T), SMAC mimetic SM-164 (S), and the pan-caspase inhibitor zVAD (Z) (herein, TSZ) (Fig. 1a). TSZ stimulation resulted in a more robust transcriptional activation of the 163 genes than TNF-α stimulation alone (Fig. 1b). Thus, we hypothesized that the degree of NF-κB recruitment might explain the increased gene transcription during TSZ stimulation. The genomic occupancy of the p65 subunit of NF-κB was therefore evaluated via chromatin immunoprecipitation sequencing (ChIP-Seq) in TNF-α-or TSZ-stimulated cells. We found TNF-α or TSZ treatment induced p65 binding in 497 different regions located near the aforementioned 163 genes. Motif analysis indicated the predicted DNA-binding motif of p65 was enriched in these genes (Fig. S1A), and further meta-analysis confirmed that TNF-α and TSZ stimulation increased the p65 ChIP-seq signal in 497 regions, though the latter induced weaker signals (Fig. 1c and Fig. 1d). As the time-course of these experiments was a full 4 h, it remained possible that some changes in p65 recruitment may have been missed. We therefore repeated our experiments with TNF-α and TSZ stimulation for 30 min; this resulted in increased p65 binding compared to the 4 h timepoint. Nonetheless, a similar trend was observed in that the p65 ChIP signal following TNF-α treatment was higher than was observed in response to TSZ treatment (Fig. 1e and Fig. S1B). In agreement with these results, the nuclear translocation of p65 did not differ significantly between TNF-α and TSZ treatment as followed by cell fractionation experiments (Fig. S1C).

We reduced the number of candidate genes in consideration from 163 to 50 genes by excluding 113 genes induced by both TNF-α and TSZ stimulation, regardless of the presence of a potent inhibitor of necroptosis, Necrostatin-1 (Nec-1). These 50 genes were substantially downregulated by Nec-1 treatment (Fig. S1D and F), meaning that they were specifically reduced when necroptosis was inhibited; however, 165 p65 ChIP-seq regions located near these genes had lower increase in p65 signals after TSZ treatment than when cells were treated with TNF-α alone (Fig. S1G). Importantly, p65 translocation after TSZ stimulation was not affected by Nec-1 treatment (Fig. 1f). Enhanced transcriptional activation of cytokine genes in response to TSZ was dramatically decreased by treatment with necroptosis inhibitors, Nec-1 and GSK’872 (Fig. 1g and h). Phosphorylation of p65, an upstream event of NF-κB transactivation, was not influenced by necroptosis inhibitors; however, IL-8 expression induced by TSZ treatment was decreased by Nec-1 treatment to similar level with TNF-induced IL-8 expression (Fig. 1i). Therefore, another mechanism, in addition to NF-κB transactivation, might contribute to transcriptional hyperactivation during necroptosis.

### TRIM28 is a transcriptional regulator that interacts kinase-active RIPK3

**Fig. 2:**
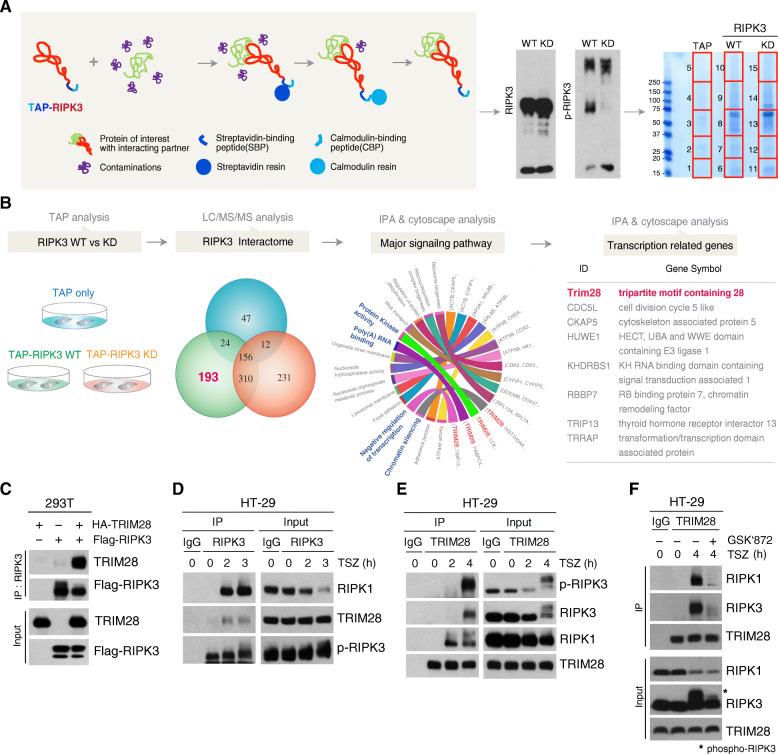
TRIM28 is a transcriptional regulator during necroptosis. **(A)** Schematic of the two step-purification workflows of RIPK3-binding proteins using a tandem affinity purification (TAP) system (left). TAP-mock, TAP-RIPK3 WT, or TAP-RIPK3-KD constructs were transiently transfected into the 293 T cells. Following 18 h of expression, the cells were lysed and subjected to two steps of purification. Validation of RIPK3 activity and its enrichment was performed by immunoblot analysis with RIPK3 or phospho-RIPK3 antibodies (middle). An aliquot of each purified sample was loaded to SDS-PAGE and stained with Coomassie brilliant blue. Potential RIPK3-binding proteins were identified by LC-MS/MS. Red square boxes indicate the excised regions of the gels subjected to LC-MS/MS analysis (right). **(B)** Venn diagram represents the overlap of proteins and unique proteins identified by LC/MS/MS among TAP-purified samples as indicated. Total 193 proteins were identified as specific RIPK3 binding proteins. Ingenuity Pathway Analysis (IPA) and cytoscape with cluego plug-in were used for bioinformatic analysis of 193 RIPK3 binding proteins and the enriched GO term/KEGG pathway are illustrated using a chord diagram. Finally, we identified eight potential RIPK3-binding proteins linked to transcriptional regulation. **(C)** 293 T cells were transfected with Flag-RIPK3 in the absence or presence of HA-TRIM28. After 24 h, cells were harvested and immunoprecipitated with RIPK3 antibodies. **(D and E)** HT-29 cells were treated with TSZ for the indicated times, and cell lysates were immunoprecipitated with RIPK3 (D) or TRIM28 antibodies (E). **(F)** HT-29 cells were pretreated with GSK’872 for 1 h and with TSZ for 4 h. Cell lysates were immunoprecipitated with TRIM28 antibodies

To understand how cytokines are produced during necroptosis, we employed tandem-affinity purification linked to mass spectrometry (TAP-MS) to identify the regulators of necroptosis-mediated transcriptional changes. The purification steps for proteins isolated in association with our TAP fusion constructs are illustrated in Fig. 2A. For the nonspecific-interaction control, we utilized a TAP-mock construct lacking any RIPK3 sequence. A TAP-RIPK3 wild-type (WT) fusion protein was used to isolate RIPK3-interacting proteins, and a mutant TAP-RIPK3 (K50A) that was enzymatically inactive (KD) acted as a control to eliminate proteins that interacted with RIPK3 that did not require its activation. After subtracting the proteins detected in the TAP-mock and TAP-RIPK3-KD, we identified 193 potential proteins that bound only to the kinase active RIPK3 WT construct (Fig. 2b). To verify the molecular functions and biological processes associated with these proteins, we performed bioinformatic analysis and grouped them according to their biological processes and molecular functions such as kinase activity, poly (A) binding, negative regulation of transcription, and chromatin silencing (Fig. 2b and Fig. S2A).

Among the potential RIPK3 binding proteins, eight were linked to transcriptional regulation (Fig. 2b), including the tripartite motif containing 28 (TRIM28), which we considered to be our most promising target as it is a transcriptional intermediary factor that acts primarily as a scaffold in several complexes for transcriptional regulation [27, 28] and is known to mediate TNF-dependent acetylation of NF-κB [29]. Therefore, we sought to determine the function(s) of TRIM28 responsible for NF-κB transcriptional activity that specifically arises when RIPK3 is activated, as happens during necroptosis. Co-immunoprecipitation verified the interaction between WT RIPK3 and TRIM28 (Fig. 2c and Fig. S2B), while the kinase-dead mutant of RIPK3 lost its activity to interact with TRIM28 (Fig. S2C). These results thus validated the results of our TAP system on an individual molecular basis. Importantly, the RIPK3 inhibitor dabrafenib (DAB) blocked the RIPK3-TRIM28 interaction (Fig. S2D), indicating the activity of the kinase was essential for its interaction. We next examined whether endogenous RIPK1/RIPK3 necrosome complexes could interact with TRIM28. As shown in Fig. 2d and e, TRIM28 was specifically recruited within necrosome complexes upon TSZ treatment; inhibition of RIPK3 activation by a different RIPK3 inhibitor, GSK’872, prevented TRIM28 interaction with necrosome components (Fig. 2f). Taken together, these results strongly suggest that TRIM28 is involved in necroptosis-mediated signaling events.

### TRIM28 antagonizes NF-κB transactivation independent of p65 chromatin occupancy

**Fig. 3:**
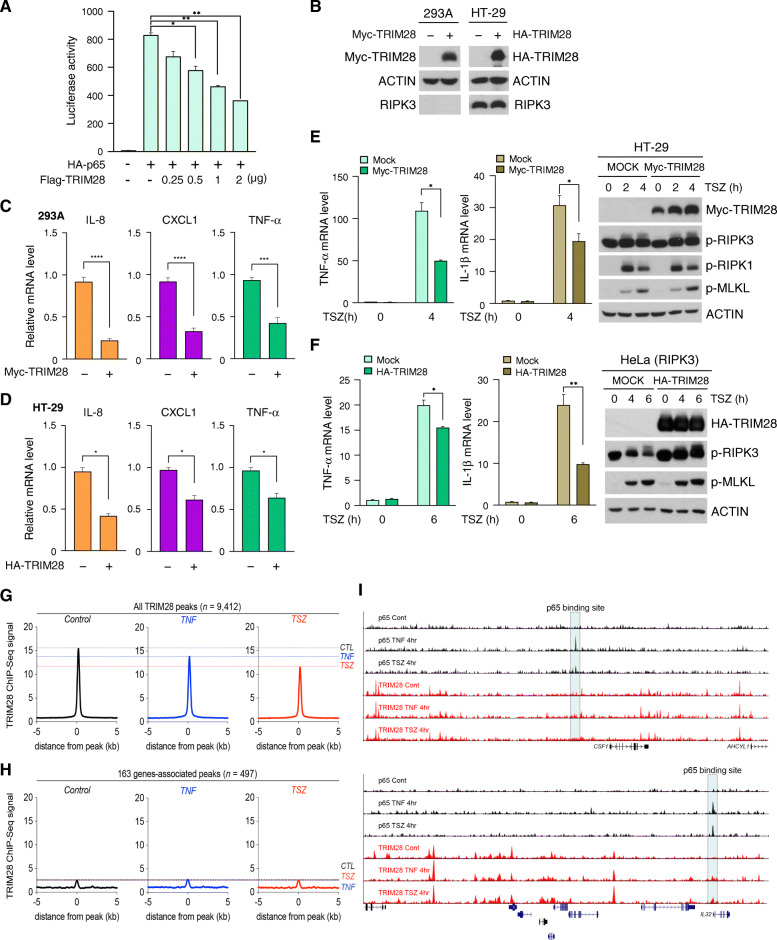
TRIM28 antagonizes NF-κB transactivation independent of p65 chromatin occupancy. **(A)** HA-p65 vector was transiently co-transfected with various doses of Flag-TRIM28 in 293 T cells. After 24 h, luciferase assays were performed, and the activity of each sample was normalized to Renilla activity. **(B)** 293A and HT-29 cells were transiently transfected with Myc-TRIM28 or HA-TRIM28 for 24 h. Cell lysates were subjected to western blotting. **(C and D)** 293A or HT-29 cells were transiently transfected with the TRIM28 vector. After 24 h, *IL-8*, *CXCL1* and *TNF-α* mRNA levels were measured by qPCR. **(E and F)** HT-29 or HeLa (RIPK3) cells were transiently transfected with the TRIM28 vector. After 24 h, cells were treated with TSZ for the indicated times. Cell lysates were subjected to western blotting (right), and mRNA levels were determined by qPCR (left). **(G)** Metagene representation of the ChIP-seq signal for TRIM28 across 9412 TRIM28-occupied regions in HT-29 cells treated with TNF-α or TSZ for 4 h. Metagenes centered on the middle of the 9412 regions and 10 Kb around the center of the regions are displayed. **(H)** Metagene representation of the ChIP-seq signal for TRIM28 across 497 regions and 163 genes-associated peaks in HT-29 cells treated with TNF-α or TSZ for 4 h. Metagenes centered on the middle of 497 regions and 10 Kb around the center of 497 regions are displayed. **(I)** ChIP-seq profiles of p65 and TRIM28 in HT-29 cells treated with TNF-α or TSZ for 4 h at the *CSF1* and *IL32* loci

Quantification of NF-κB-driven luciferase reporter activity indicated that TRIM28 antagonized p65-dependent transactivation in a dose-dependent manner (Fig. 3a). Correspondingly, overexpression of TRIM28 reduced the endogenous mRNA levels of known NF-κB targets, such as *IL-8*, *CXCL1* and *TNF-α* in 293A and HT-29 cells (Fig. 3b-d). TRIM28 overexpression also impaired TSZ-mediated cytokine production in HT-29 and HeLa-RIPK3-expressing cells, whereas the phosphorylation of necrosome components RIPK1, RIPK3, and MLKL in response to TSZ was unaffected (Fig. 3e and f). These results suggest that TRIM28 functions as a negative regulator of NF-κB transcription activity that acts downstream of formation of the necrosome complex. We speculated that TRIM28 repressed the NF-κB-driven promoter activity of cytokine genes in the absence of necroptosis. While ChIP-seq analysis of TRIM28 showed that both TNF-α and TSZ stimulation reduced the chromatin binding of TRIM28 in 9412 TRIM28-bound regions (Fig. 3g), p65-binding signals were not detected in these regions (Fig. 3h and i), indicating that TRIM28 does not interfere p65-occupied chromatin regions. Our data therefore suggest that TRIM28 prevents NF-κB-independent cytokine production during resting states and that the inactivation of TRIM28, along with NF-κB, promotes necrosome-induced transcriptional changes.

### RIPK3 activation induces TRIM28 phosphorylation at serine 473

**Fig. 4:**
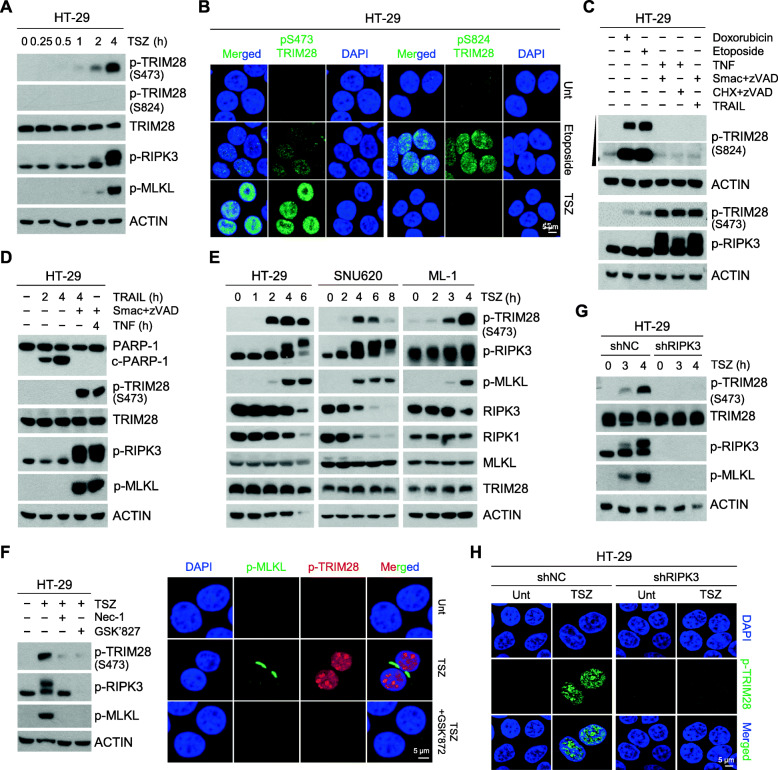
RIPK3 activation induces TRIM28 phosphorylation at serine 473. **(A)** HT-29 cells were treated with TSZ in a time-dependent manner. Cell lysates were analyzed by western blotting. **(B)** TSZ-treated HT-29 cells were stained with S824 and S473 phospho-TRIM28 antibodies and analyzed by confocal fluorescence microscopy (green: S824 or S473 phospho-TRIM28; blue: DAPI). **(C)** HT-29 cells were treated for 4 h with doxorubicin (2 μM), etoposide (100 μM), TSZ, TNF-α + CHX (5 μg/ml) + zVAD, or TRAIL (100 ng/ml) + SZ. **(D)** HT-29 cells were treated with TRAIL, TRAIL+ SZ, or TSZ for the indicated times, and cell lysates were analyzed by western blotting. **(E)** HT-29, SNU620, and ML-1 cells were treated with TSZ for the indicated times, and cell lysates were analyzed by western blotting. **(F)** HT-29 cells were pretreated with Nec-1 and GSK’872 for 1 h and with TSZ for 4 h. Cell lysates were analyzed by western blotting (left). Cells were also stained and visualized by confocal fluorescence microscopy (green: phospho-MLKL; red: S473 phospho-TRIM28; blue: DAPI) (right). **(G)** HT-29 cells stably expressing RIPK3 shRNA or the non-silencing control were treated with TSZ for the indicated times. **(H)** Cells from (G) were treated with TSZ for 4 h, stained, and visualized by confocal fluorescence (green: S473 phospho-TRIM28; blue: DAPI)

To investigate how necrosome formation may induces transcriptional changes via TRIM28 inactivation, we examined TSZ stimulation-dependent changes upon TRIM28 phosphorylation, which is known to reduce its co-repressor activity [30–32]. TRIM28 is phosphorylated at S824 and S473 in response to DNA damage [33–35]. Phosphorylation at S824 regulates the expression of genes mainly involved in the cell cycle and apoptosis, whereas S473 phosphorylation initiates the transcriptional derepression of IFN-β, IL-8, and IL-6 cytokines [32, 36]. The genotoxic agents, doxorubicin and etoposide indeed induced TRIM28 phosphorylation at both S824 and S473 in a dose- and time-dependent manner (Fig. S3A), and the DNA damage response to these was confirmed by the phosphorylation status of ataxia-telangiectasia mutated (ATM) kinase and histone H2A.X (γH2AX), which are involved in DNA-damage sensing pathways (Fig. S3B). Surprisingly, however, following TSZ stimulation, we only detected TRIM28 phosphorylation at S473 in a time-dependent manner but not at S824 in HT-29 cells (Fig. 4a). TSZ stimulation-mediated TRIM28 phosphorylation at S473 was also detected in HeLa (RIPK3) cells (Fig. S3C). Immunofluorescence staining analysis also showed that TSZ stimulation remarkably induced the phosphorylation of TRIM28 at the S473, but not S824 (Fig. 4b and Fig. S3D and E).

TRIM28 at S473 was markedly phosphorylated in response to both TNF and TRAIL in the presence of SMAC mimetic and zVAD (Fig. 4c and d), conditions that lead to the phosphorylation of RIPK3 and MLKL and to necroptosis. However, when TRAIL alone (in the absence of Smac mimetic and zVAD) was used to initiate cell death in the HT-29 cells, cell death occurred through an apoptotic, rather than necroptotic mechanism, as indicated by PARP-1 cleavage and lack of RIPK3 and MLKL phosphorylation (Fig. 4d). Importantly, when apoptosis was induced by TRAIL, TRIM28 phosphorylation at S473 did not occur (Fig. 4d), indicating that TRIM28 phosphorylation at S473 is likely dependent on RIPK3 activation. TRIM28 S473 phosphorylation was further observed in multiple cell lines treated with TSZ, including SNU620, ML-1, NIH3T3, and RAW264.7 cells (Fig. 4e and Fig. S3F). However, inhibition of RIPK1/RIPK3 kinase activity by necroptosis inhibitors, i.e., Nec-1, GSK’872, and dabrafenib (DAB), abolished S473 phosphorylation (Fig. 4f, Fig. S3G, and H). Depletion of RIPK3 via shRNA knockdown also eliminated this TRIM28 phosphorylation (Fig. 4g, h, and Fig. S3I-L). These results indicate that TRIM28 phosphorylation at S473 is RIPK3-dependent and suggest that RIPK1/RIPK3 activation induces TRIM28 phosphorylation at S473, which may play an important role in the regulation of transcriptional activity.

### RIPK3-dependent phosphorylation of TRIM28 induces enhanced transcriptional activity

**Fig. 5:**
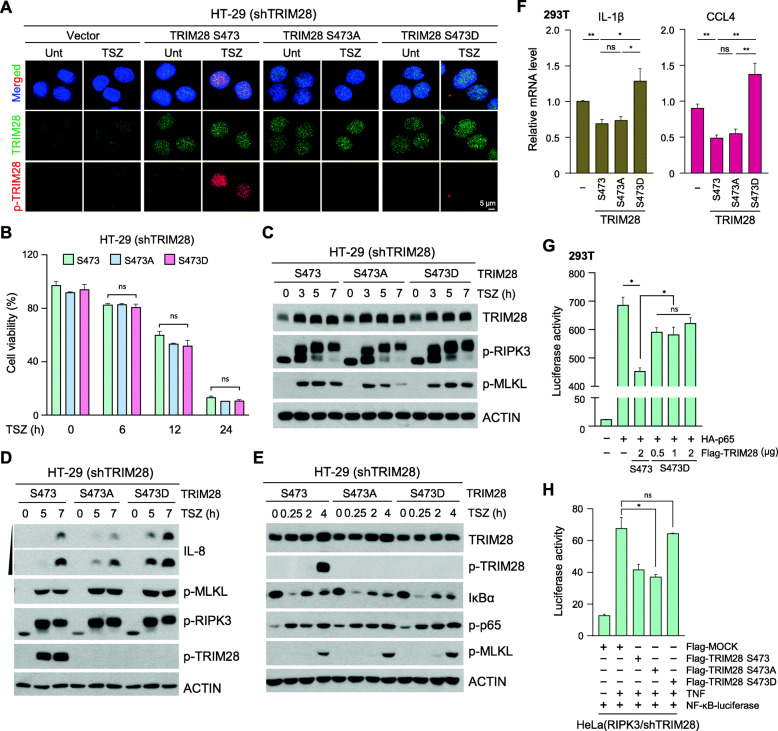
TRIM28 S473 phosphorylation induces enhanced transcriptional activity. **(A)** TRIM28-knockdown HT-29 cells were reconstituted with the mock-vector or TRIM28 S473 mutants (TRIM28 S473 wild-type, TRIM28 S473A, and TRIM28 S473D). Cells were treated with TSZ for 4 h, stained, and visualized by confocal fluorescence microscopy. (green: TRIM28; red: S473 phospho-TRIM28; blue: DAPI). **(B-E)** Cells from (A) were treated with TSZ at the indicated times, and cell viability was determined by MTT assay (B). Cell lysates were analyzed by western blotting (C, D, and E). **(F)** 293 T cells were transiently transfected with Flag-TRIM28 S473 WT or the S473 mutants. After 24 h, *IL-1β* and *CCL4* mRNA levels were measured by qPCR. **(G)** 293 T cells were transiently transfected with HA-p65, Flag-TRIM28 S473 WT, and various doses of Flag-TRIM28 S473D. After 24 h, luciferase activity was measured. **(H)** TRIM28-knockdown HeLa cells stably expressing RIPK3 were transiently transfected with Flag-TRIM28 S473 WT or S473 mutants. After 24 h, cells were treated with TNF-α for 6 h, and luciferase activity was measured

Our findings to this point suggested that RIPK3 activation-induced TRIM28 phosphorylation at S473 plays an important biological function during necroptosis. We next stably expressed different versions of TRIM28 including WT, a phosphorylation-deficient S473A mutant, and a phosphorylation-mimicking S473D mutant in cells in which endogenous TRIM28 was stably knocked down. These cells were treated with TSZ to activate RIPK3 and necroptosis. Immunofluorescence staining showed that S473 phosphorylation occurred only in wild-type TRIM28-expressing cells (Fig. 5a). Importantly, cells expressing S473A and S473D mutants had no alteration in cell death sensitivity (Fig. 5b) or RIPK3/MLKL phosphorylation in response to TSZ stimulation (Fig. 5c and Fig. S4A). However, the expression of TRIM28 S473D was enhanced TSZ-induced IL-8 production as well as *IL-1β* mRNA relative to the other TRIM28 mutants (Fig. 5d, Fig. S4B and C), suggesting that TRIM28 phosphorylation at S473 regulates transcriptional activity. TRIM28 phosphorylation status did not obviously influence canonical upstream NF-κB signaling events, such as IκBα degradation or p65 phosphorylation (Fig. 5e and Fig. S4D), suggesting that S473 phosphorylation of TRIM28 alters transcription once p65 is localized to the nucleus. As expected, expression of the TRIM28 S473 wild-type and the TRIM28 S473A mutant, but not TRIM28 S473D, suppressed cytokine production (Fig. 5f and Fig. S4E).

In accordance with our data showing that TRIM28 antagonizes the p65-dependent transactivation as evidenced by the NF-κB-driven luciferase reporter activity (see Fig. 3A), we hypothesize that phosphorylation of TRIM28 at S473 disturbs its transcriptional repressor activity. Phosphorylation of S473 has been shown to impair the physical interaction between TRIM28 and HP1 on the chromatin [30], but as TRIM28 does not directly bind to DNA in a sequence-specific manner [37], its repression of gene expression requires its interaction with other transcription factors, including STAT3, IRF5, and NF-κB [29, 38, 39]. Consequently, we found that the TRIM28 S473D mutant has a weaker binding affinity to p65 than the S473A mutant, indicating that the phosphorylation status at S473 likely controls the interaction of TRIM28 with transcription factors (Fig. S4F). Consistent with a previous study [34], the TRIM28 S473D mutant lost its interaction activity with HP1α (Fig. S4G). Based on this, we examined whether S473 phosphorylation would directly affect its ability to repress NF-κB activation. Cells were co-transfected with TRIM28 S473A mutant or the phosphor mimetic S473D mutant and analyzed for NF-κB-driven luciferase reporter activity. The TRIM28 S473 WT antagonized the p65-dependent transactivation, whereas the S473D mutant did not (Fig. 5g and Fig. S4H). TNF-α or TSZ-stimulation-mediated NF-κB-driven luciferase reporter activity was also not affected in the S473D mutant (Fig. 5h, Fig. S4I, and J). These data suggest that the RIPK3-dependent TRIM28 S473 phosphorylation induces the inactivation of its co-repressive function.

### TRIM28 negatively regulates necrosome-induced cytokine production

**Fig. 6:**
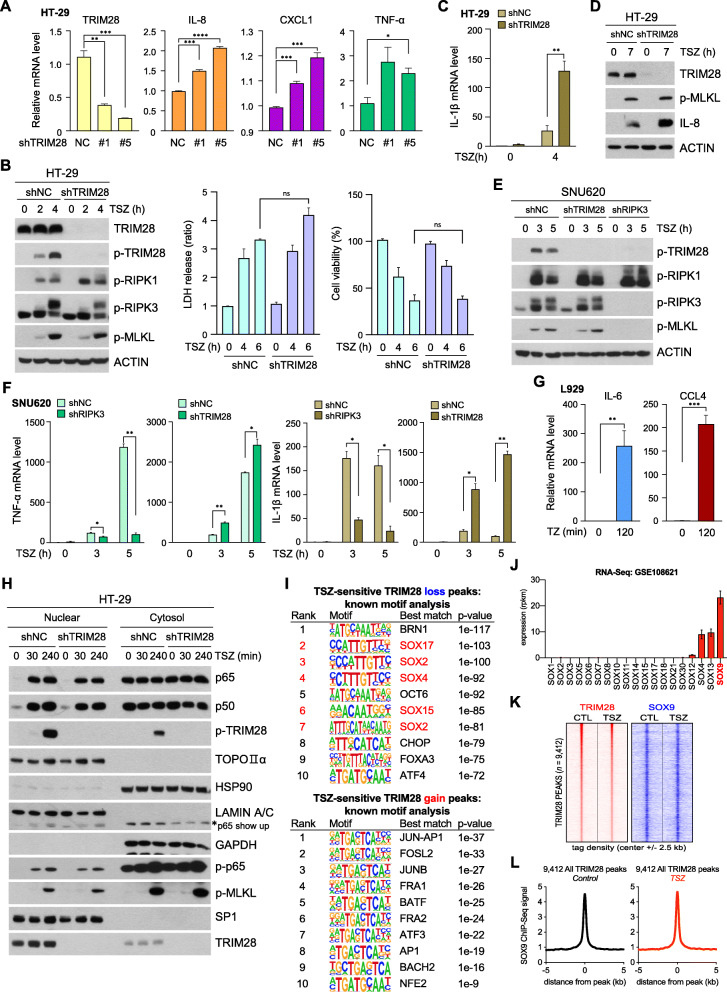
TRIM28 negatively regulates necrosome-induced cytokine production. **(A)** mRNA expression levels of HT-29 cells expressing TRIM28 shRNA or the non-silencing control were analyzed by qPCR. **(B)** Cells from (A) were treated with TSZ for the indicated time points and analyzed by western blotting (left panel), LDH leakage (middle), and MTT assay (right panel). **(C and D)** Cells were treated with TSZ for indicated times. Their mRNA levels were analyzed by qPCR (C), and cell lysates were subjected to western blotting (D). **(E and F)** SNU-620 cells stably expressing TRIM28 shRNA or RIPK3 shRNA were treated with TSZ in a time-dependent manner. Cell lysates were analyzed by western blotting (E), and mRNA levels were analyzed by qPCR (F). **(G)** L929 cells were treated with TZ for 2 h, and *IL-6* and *CCL4* mRNA levels were analyzed by qPCR. **(H)** HT-29 cells expressing TRIM28 shRNA or a non-silencing control were fractionated into nuclear and cytosol fractions after the indicated treatments. Fractionation samples were analyzed by western blotting. **(I)** Representation of motifs enriched at TSZ-sensitive TRIM28 loss peaks (top) and TSZ-sensitive TRIM28 gain peaks (bottom). Known motif analysis was performed using HOMER; the top 10 ranked motifs are shown with their *p*-values. **(J)** RNA-seq-based expression of SOX family proteins in HT-29 cells (GSE108621). **(K)** Density plot of TRIM28 and SOX9 ChIP-seq datasets centered on 9412 TRIM28-occupied regions in HT-29 cells treated with DMSO or TSZ for 4 h. Each row represents a single peak. **(L)** Metagene representation of the ChIP-seq signal for SOX9 across 9412 TRIM28-occupied regions in HT-29 cells treated with TSZ for 4 h. Metagenes centered on the middle of 9412 regions and 10 Kb around the center of the TRIM28-occupied regions are displayed

Western blotting experiments in HT-29 cells showed that stable shRNAs targeting TRIM28 decreased the amount of TRIM28 protein without affecting the RIPK1 and RIPK3 proteins (Fig. S5A). Notably, TRIM28 knockdown resulted in increases in cytokine mRNA expression (Fig. 6a); however, RIPK1/RIPK3/MLKL phosphorylation and cell death upon TSZ stimulation were unaffected (Fig. 6b, Fig. S5B, and C). More importantly, TSZ stimulation in TRIM28 depleted cells resulted in increased amounts of *IL-1β*, *TNF-α*, and *CCL4* mRNA (Fig. 6c and Fig. S5C). Cytokines were further quantitated on a protein level to show that depletion of TRIM28 resulted in increases in cytokine secretion (Fig. S5D). We confirmed an increase in IL-8 upon TSZ stimulation in TRIM28-depleted cells by western blotting (Fig. 6d).

Similar results were obtained in SNU620 and HeLa (RIPK3) cells. Consistently, TRIM28 knockdown enhanced cytokine production without changes of necroptosis signaling, but depletion of RIPK3 abolished both TRIM28 phosphorylation and cytokine production (Fig. 6e, f, and Fig. S5E and F). Similar to human cancer cell lines, mouse fibrosarcoma cell line, L929 cells also showed necroptosis-dependent TRIM28 phosphorylation (Fig. S5G) and increased mRNA level of immunostimulatory cytokine (Fig. 6g and Fig. S5H). Analysis of RIPK3 activation-dependent transcriptional changes in Nec-1 pretreated cells revealed the downregulation of *IL-6* and *CCL4* (Fig. S5I). However, TRIM28 depletion had no significant effect on p65 phosphorylation or nuclear translocation (Fig. 6h and Fig. S5J and K). In agreement with the non-overlapping pattern of TRIM28 and NF-κB genomic occupancy shown in Fig. 3I, our results suggest that TRIM28 does not directly repress NF-κB activity on chromatin, functioning independently perhaps through chromatin co-repressors.

The inhibitory function of TRIM28 on cytokine production was demonstrated in the resting stage (Fig. 3c, d, and 6a), suggesting that chromatin-bound TRIM28 may be involved in cytokine production by interacting with other transcription factors. DNA motif enrichment analysis at TRIM28-depleted genomic regions upon TSZ stimulation showed that five of the ten significantly enriched motifs were related to SOX transcription factors (Fig. 6i upper panel). Genomic sites where TRIM28 binding is increased upon TSZ stimulation lacked SOX-related motifs (Fig. 6i lower panel), suggesting that the occurrence of SOX motifs is specific to regions where TRIM28 was displaced upon TSZ stimulation. RNA-seq analysis of data from HT-29 cells showed that SOX9 was the most highly expressed among all the Sox family transcription factors (Fig. 6j), which prompted us to determine SOX9 genomic occupancy via ChIP-seq assay. From our SOX9 ChIP-seq experimental results, we found that SOX9 ChIP-seq signals were aligned to the center of TRIM28-lost regions regardless of TSZ treatment (Fig. 6k, l, and Fig. S6A), suggesting that reduced TRIM28 binding activity with chromatin during TSZ stimulation is likely to be related to SOX9 transactivation contributing to cytokine hyperproduction, along with NF-κB. In particular, we found that additional SOX9 is recruited to the promoter of cytokine genes together with NF-κB under TSZ stimuli (Fig. S6B), further emphasizing the role of TRIM28 in negatively regulating the activity of immune cytokine-related transcription factors.

### Derepression of TRIM28 leads to synthesis of immunostimulatory cytokines

**Fig. 7:**
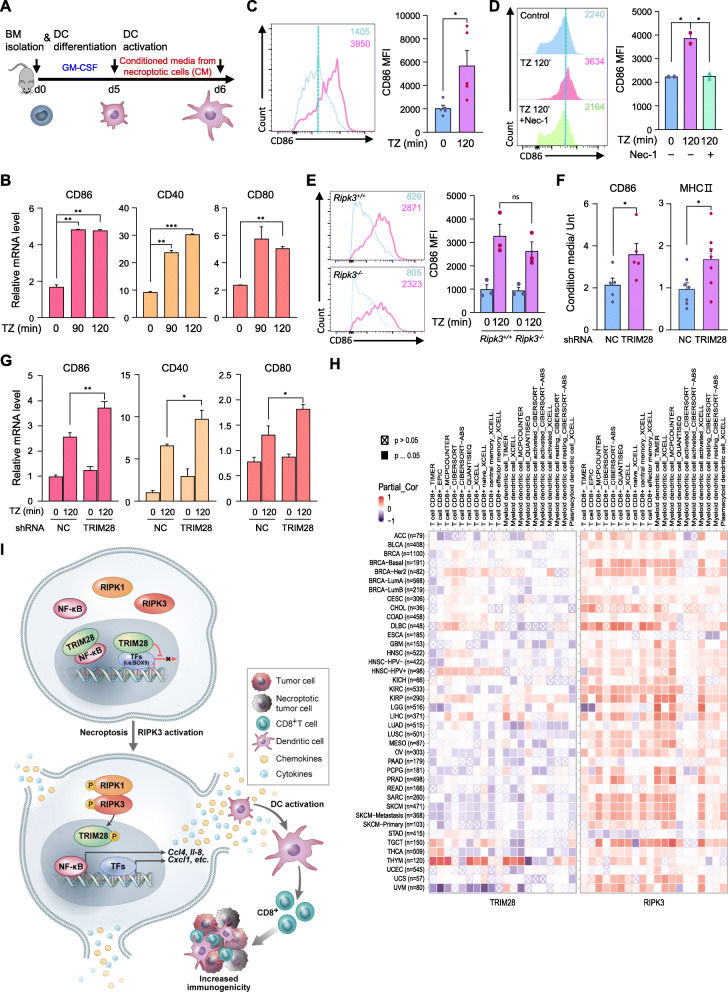
Derepression of TRIM28 leads to increased synthesis of immunostimulatory cytokines. **(A)** Bone marrow-derived monocyte (BM) differentiated into dendritic cells (DC) upon culture with GM-CSF for 5 d. Conditioned medium from necroptotic cells (CM) or control cells was added for further activation. **(B)** DCs were treated with CM for 16 h, and *CD86*, *CD40*, and *CD80* mRNA levels were analyzed by qPCR. **(C)** DCs were treated with CM for 16 h, and CD86 expression levels were analyzed by FACS. **(D)** DCs were treated with CM from L929 cells, which were treated with TZ for 120 min in the absence or presence of Nec-1 (CM), for 16 h. FACS analysis was performed. **(E)** DCs from RIPK3 WT or RIPK3 KO mice were treated with CM. CD86 expression was analyzed by FACS. **(F and G)** DCs were treated with CM from L929 cells stably expressing TRIM28 shRNA or the non-silencing control for 16 h. CD86 and MHC II expression was analyzed by FACS (F), and *CD86*, *CD40* and *CD80* mRNA levels were analyzed by qPCR (G). **(H)** The heatmap table shows the association between *TRIM28 or RIPK3* and tumor-infiltrating level of multiple types of CD8^+^ T cell or DC, and that is estimated by six algorithms across cancer types in the TCGA database. Red indicates a statistically significant positive association, and blue indicates a statistically significant negative association. **(I)** Schematic of proposed RIPK3/TRIM28 activation-mediated immunostimulatory cytokine production

In contrast to the TNF-α-mediated transcriptional activation, our data so far indicate that necroptosis-induced cytokine production is accompanied by the inactivation of TRIM28 co-repressive function. Since cytokine upregulation is more physiologically relevant to immune system cells, we chose to examine it in more detail in cells from the mouse immune system. We first isolated bone marrow-derived monocytes (BM) and cultured them with granulocyte-macrophage colony-stimulating factor (GM-CSF) for 5 ~ 6 days to differentiate immature dendritic cells (iDCs), which were further cultured with cellular supernatants from cells in which necroptosis and RIPK3 activation was initiated (Fig. 7a, Fig. S7A and B). Upon culture with supernatant from these cells, *CD86*, *CD40*, and *CD80* mRNA levels were upregulated in DCs (Fig. 7b) and cell surface markers CD86 and MHC II were also increased in DCs (Fig. 7c and Fig. S7C). We also found that the increase in DC surface markers by treatment with the active supernatants from necroptotic cells was significantly reduced when incubating iDCs with the supernatant of Nec-1 pretreated cells. (Fig. 7d).

We hypothesized that the cellular supernatant from RIPK3 activation-induced cells contain necroptosis-inducing agents such as TNF-α and zVAD and that these agents could induce necroptosis to increase cytokine production in iDCs. To verify this, we cultured iDCs from both *Ripk3*^*+/+*^ and *Ripk3*^*−/−*^ mice with cellular supernatants from RIPK3 activation-induced cells, but found no difference in DC activation and cell death (Fig. 7e and Fig. S7D). Dendritic cells from both *Ripk3*^*+/+*^ and *Ripk3*^*−/−*^ mice showed similar levels of cell-surface markers, CD11c ^+^ (Fig. S7E). These data indicate that RIPK3 activation-dependent upregulation of cytokine production contributes to DC activation. Importantly, TRIM28 knockdown further increased necroptosis-induced *IL-6* production and under the same conditions, cell-surface markers CD86 and MHC II were potently increased (Fig. S7F and Fig. 7f). In addition, cellular supernatant from these cells enhanced *CD86, CD40*, and *CD80* mRNA expression in DCs (Fig. 7g). Bioinformatics analysis of the TCGA database strongly supports these in vitro results, as it showed that the expression of RIPK3 has a significant positive association with the tumor-infiltrating populations of several types of CD8^+^ T cells or DCs in various tumor type (Fig. 7h). This was a similar trend as IFN-β, a well-known factor that supports anti-tumor immunity, which was used as a positive control (Fig. S7G). However, the expression of TRIM28 had a significant negative association with the tumor-infiltrating immune cell populations, consistent with our hypothesis (Fig. 7h). Taken together, our data indicate that the activation of RIPK1/RIPK3 within the tumor microenvironment promotes the synthesis of immunostimulatory cytokines, thereby activating anti-tumor immunity (Fig. 7i).

## Discussion

The potential therapeutic value of necroptosis has been recognized in the treatment of various inflammatory diseases including cardiovascular disease, infectious disease, renal disease, bowel disease, and neurodegenerative diseases [6, 40–43]. In particular, it has been shown that the activation of RIPK1/RIPK3 during necroptosis not only induces the release of DAMPs, following the loss of cell membrane integrity but also releases cytokines produced via active transcription, resulting in increased immunogenicity; creates an inflamed microenvironment to recruit innate immune cells and support them. Several studies suggested NF-κB as the main transcription factor in this cytokine production [9, 11, 12]. However, another study showed NF-κB to be dispensable for necroptosis-induced immunogenicity [44]. Like this, the detailed mechanism by which necroptosis regulates the inflammatory transcription program is largely unknown. Here, we demonstrate that RIPK3 regulates the expression of inflammatory cytokines via both NF-κB-dependent and -independent transactivation pathways and TRIM28 is a key negative regulator required to fine-tune the necroptosis-mediated inflammatory transcription program.

TRIM28 is known as a genomic co-repressor and a negative immune regulator of cytokine production in response to various immune stimuli [32]. Its phosphorylation has been suggested to inhibit its repression of certain gene subsets [32, 35]. Consistent with previous reports, we found that necroptosis-induced phosphorylation of TRIM28 at serine 473 inactivates its co-repressive function, thus contributing to the de novo synthesis of immunostimulatory cytokines in dying cells. Upon mutation of S473 to aspartic acid (S473D) to mimic constitutive phosphorylation, TRIM28 loses its repressive effect on cytokine genes expression involving both NF-κB-dependent and -independent transactivation. Phosphorylation of TRIM28 at serine 473 attenuates NF-κB-binding of TRIM28 and may contribute increased NF-κB-dependent transactivation. In addition to NF-κB-dependent transactivation by TRIM28, RIPK3-mediated sequestration of TRIM28 from the chromatin can release repression on SOX9 transcriptional activity. SOX9-mediated repression of its target genes through TRIM28 was recently reported [45] and other SOX family members were also shown to correlate with activation or repression of TRIM28 target genes [46, 47]. From SOX9 ChIP-Seq data, we can speculate that reduced TRIM28 binding from chromatin during TSZ stimulation may turn on SOX9-mediated transactivation to express cytokines. Although phosphorylation of TRIM28 at S824, which has been previously shown to actively induce chromatin relaxation, is a critical modification for regulation of gene expression [48], we did not detect phosphorylation of TRIM28 at S824 in response to TSZ, suggesting that RIPK3-mediated TRIM28 phosphorylation at S473 leads to the loss of TRIM28 co-repressive functions. Phosphorylation of TRIM28 has been reported to repress sumoylation of TRIM28 suggesting that phosphorylation-dependent inhibition prevents SUMO-mediated heterochromatin formation [36]. Based on this, there is the possibility that other post-translational modification (PTM) could be involved in transcriptional repression.

Recently, more attention has been paid to necroptosis as an attractive form of cell death, with the realization this particular cell death mode efficiently initiates immunogenic cell death (ICD), providing potential therapeutic strategies to increase immunogenicity and improve the efficacy of T-cell-based therapies in “immune desert” solid tumors (i.e., tumors without immune cell infiltration) [49]. Indeed, Snyder et al. proposed that intratumoral necroptosis through RIPK1/RIPK3 activation induces an anti-tumor effect via a systemic immune response [12]. In this study, ectopic activation of RIPK3 promoted tumor antigen loading by tumor APCs (antigen presenting cells) associated with enhanced CD8^+^ T cell-mediated anti-tumor responses, which synergized with α-PD-1 co-administration to promote tumor clearance. In addition, Yatim et al. demonstrated that necroptotic cells induce immune stimulation, which subsequently leads to DC maturation for CD8^+^ T cell cross-priming [11]. These studies suggest that activation of RIPK1/RIPK3 within the tumor microenvironment enhances DC- and CD8^+^ T cell-mediated anti-tumor immunity. Our findings strongly support these previous studies by demonstrating that the RIPK1/RIPK3 signaling axis initiates TRIM28 derepression that promotes DC maturation; TRIM28 overexpression impairs necroptosis-mediated cytokine production. Consistently, analysis performed across all TCGA tumors shows that RIPK3 has a positive correlation and TRIM28 has a negative correlation with CD8^+^ T cells and DCs infiltrating tumor tissues, while TRIM28 has a negative correlation (Fig. 7h), thus implying that the deregulation of TRIM28 by activated-RIPK3 can enhance immunogenicity in the tumor and could be an important combination strategy with current T-cell-based immunotherapy.

Furthermore, the level of RIPK3 and TRIM28 in the tumor could be an indicator for the applicability of ICD-based immunotherapy in the selection for therapeutic approaches. Our TCGA analysis showed increased level of TRIM28 expression in various cancer types compared to normal tissues (Fig. S8A), which is consistent with previous studies that reported that TRIM28 expression is significantly higher in gastric and breast cancer tissues than their corresponding normal tissues and that downregulation of TRIM28 inhibits tumor growth and metastasis in mouse xenograft models [20, 50]. From the public database, we found that lower TRIM28 mRNA expression was associated with significantly higher overall survival (OS) of ≥150 months in gastric cancer patients (Fig. S8B). Correlation between higher *RIPK3* mRNA expression and OS was not statistically significant in gastric cancer patients as whole in this database; however, it was significant in later-stage (stage 3) gastric cancers (Fig. S8C). Furthermore, when we examined RIPK3 protein expression by histopathological measurement in our own cohort of general gastric cancer patients (*N* = 338), we found high RIPK3 protein expression was correlated with overall survival (OS, *P* = 0.048) and progression free survival (PFS, *P* = 0.044) in these patients (Fig. S8D). In this same cohort, tissue samples with high RIPK3 expression show strong reactivity to anti-CD8 and anti-Granzyme B antibodies (Fig. S8E), implicating the involvement of RIPK3 in regulating the anti-tumor microenvironment. Considering that high levels of RIPK3 expression and low level of TRIM28 expression in human gastric cancer tumors correlate with improved gastric cancer patient survival, we propose that patients who have a high level of TRIM28 expression in tumor tissue will get a beneficial effect with an improved level of RIPK3 activation using intra-tumoral treatment of RIPK3 agonist in the case of high expression of RIPK3 and adeno-associated viruses (AAVs)-mediated delivery of constitutively-active RIPK3 in case of little or no expression of RIPK3. Treatment with 5-aza-2′-deoxycidine may be an alternative to increase RIPK3 expression in the latter group, as we have previously shown some cancer cell lines and tumors respond favorably to the compound in restoring RIPK3 expression [51].

Our study strongly supports high potential of necroptosis as an ICD to infuse immunogenicity into immune-depleted tumors by providing a detailed underlining mechanism. However, the complicated crosstalk of upstream signals limits the applicability of necroptosis as an ICD for cancer immunotherapy. Therefore, future studies are needed to develop more clinically applicable strategies to modulate the specific activation of necroptosis modulators such as RIPK1, RIPK3, MLKL and TRIM28.

## Conclusion

We identified Tripartite Motif Protein 28 (TRIM28) as a co-repressor downstream of RIPK3 kinase activity that regulates transcriptional activity during necroptosis. RIPK3 activation inhibits the chromatin binding activity of TRIM28 leading to the transcriptional activation of cytokines, which then promotes immunoregulatory processes, such as dendritic cell maturation and further cytokine production, which then contribute to anti-cancer responses. Thus, RIPK3 activation-dependent regulation of TRIM28 in cancer cells is likely to significantly contribute to a robust cytotoxic anti-tumor immunity.

## Supplementary Information

**Additional file 1.**

## Data Availability

***Lead Contact*** Further information and requests for resources and reagents should be directed to and will be fulfilled by the Lead Contact, You-Sun Kim (yousunkim@ajou.ac.kr). ***Materials Availability*** Plasmids generated in this study are available upon request to the lead contact. This study did not generate new unique reagents.

## Abbreviations

CTL: Cytotoxic CD8^+^ T cell
DAB: Dabrafenib
DAMPs: Damage-associated molecular patterns
GM-CSF: Granulocyte-macrophage colony-stimulating factor
HP1: Heterochromatin protein 1
ICD: Immunogenic cell death
iDC: Immature dendritic cells
MLKL: Mixed lineage kinase domain-like pseudokinase
Nec-1: Necrostatin-1
NF-κB: Nuclear factor kappa-light-chain-enhancer of activated B cells
RIPK3: Receptor-interacting protein kinases 3
SOX9: SRY-Box Transcription Factor 9
TAP-MS: Tandem-affinity purification linked to mass spectrometry
TRIM28: Tripartite motif containing 28
