# Supplementary material for: RIPK3 activation induces TRIM28 derepression in cancer cells and enhances the anti-tumor microenvironment
Source: Mol Cancer. 2021 Aug 21;20:107. doi: 10.1186/s12943-021-01399-3 (PMC8379748; doi:10.1186/s12943-021-01399-3)
Supplement: Supplementary file 1 — Additional file 1. [file 12943_2021_1399_MOESM1_ESM.pdf]

Supplementary Figure 1.

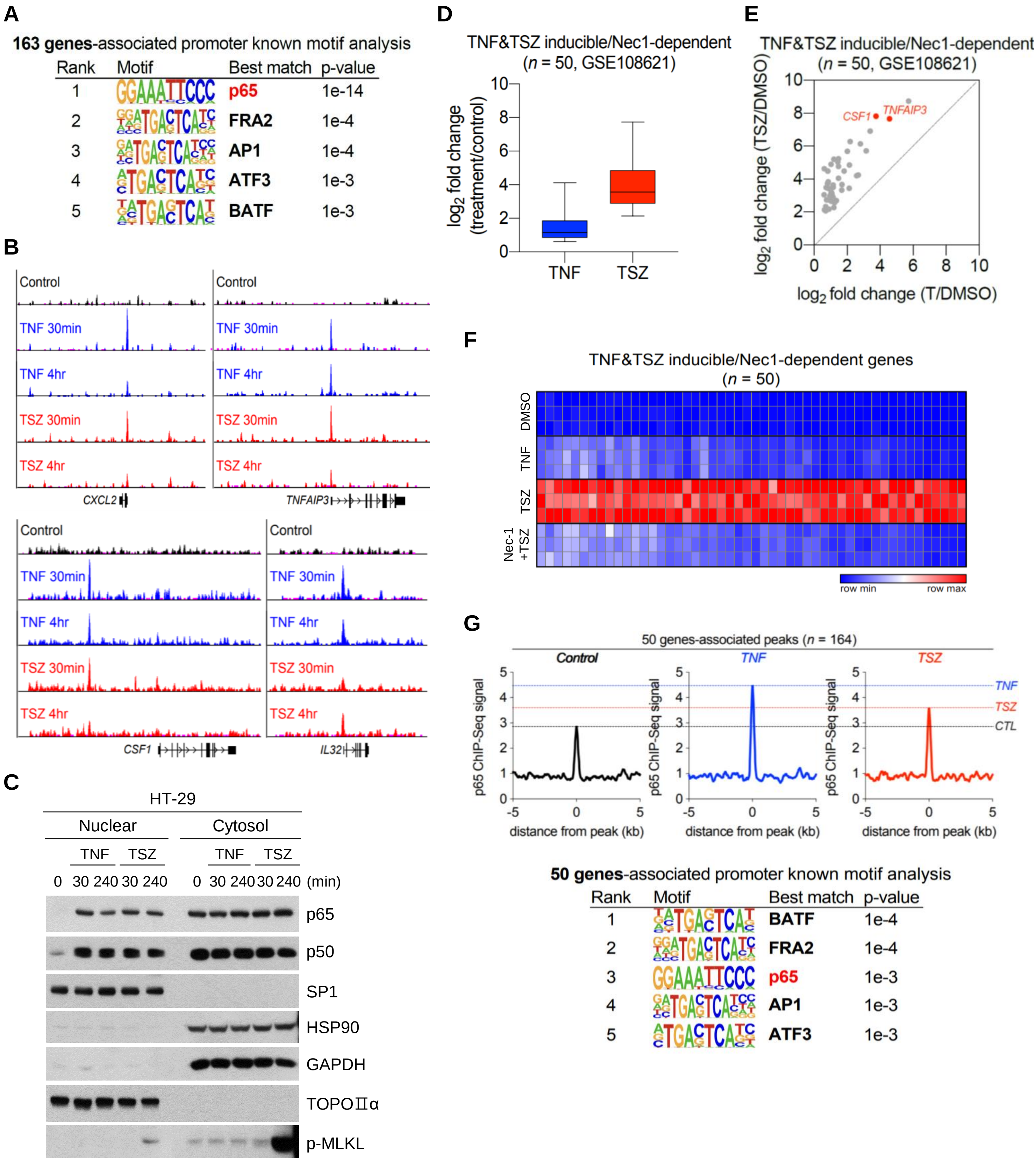

Supplementary Figure 2.

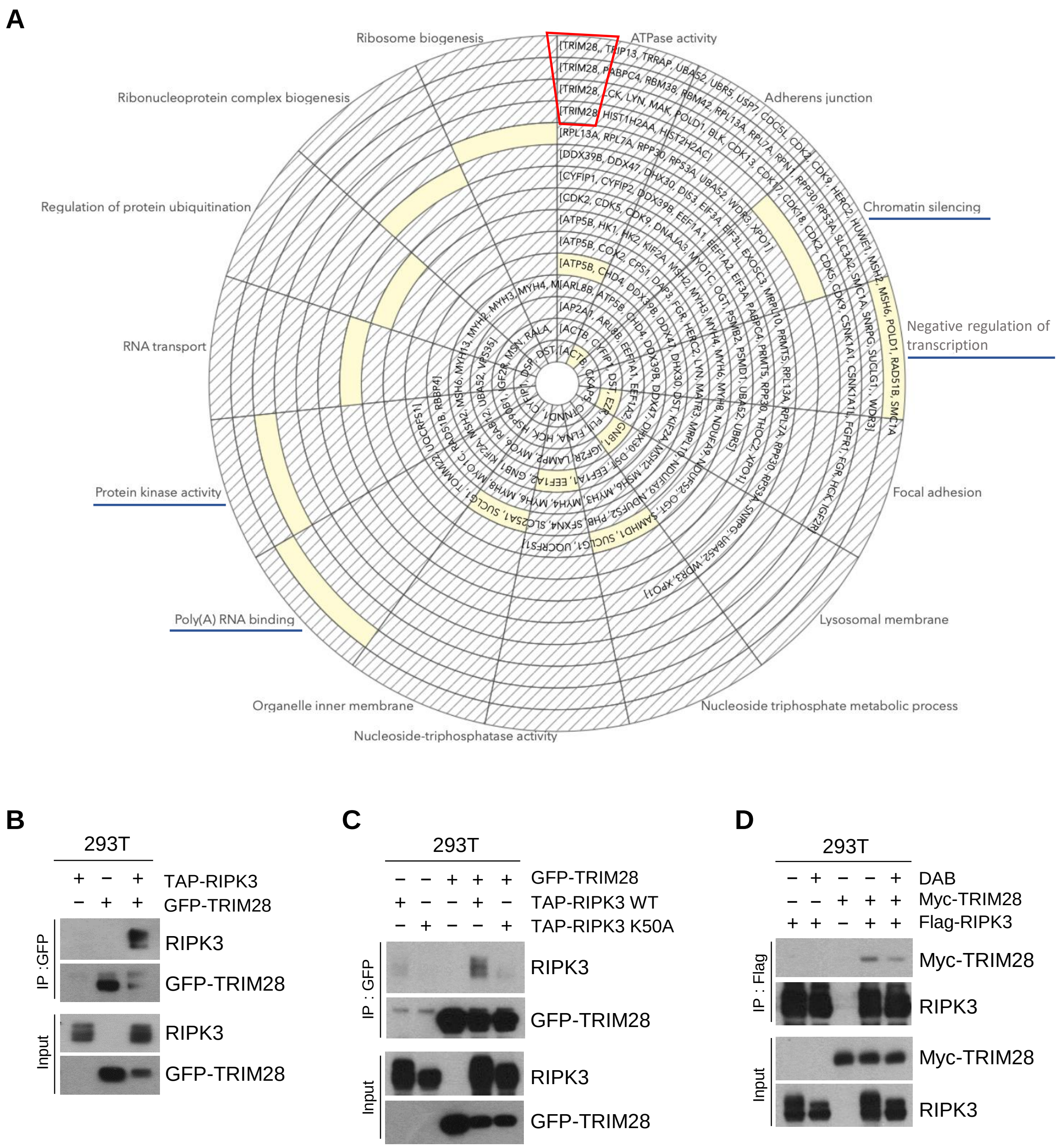

Supplementary Figure 3.

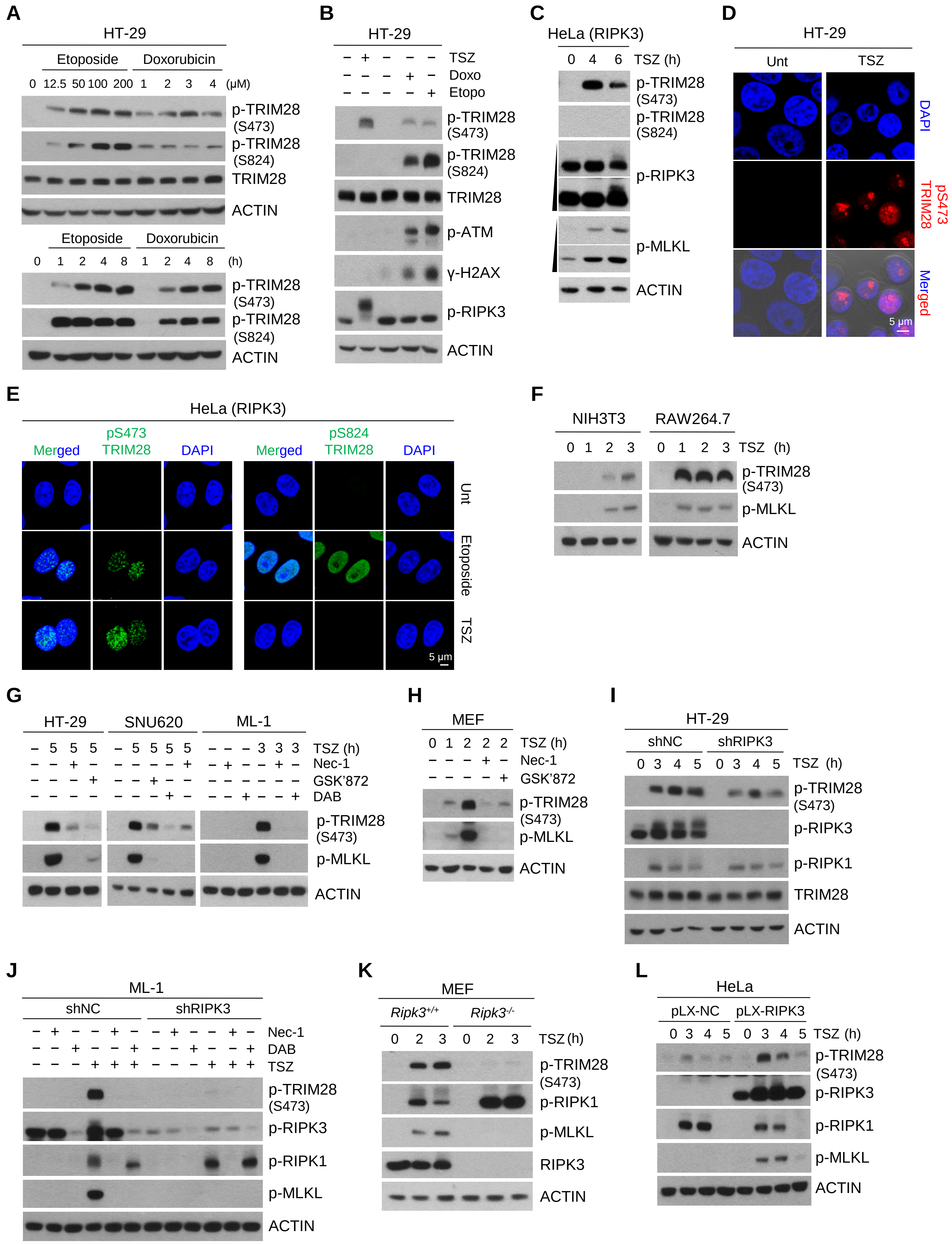

Supplementary Figure 4.

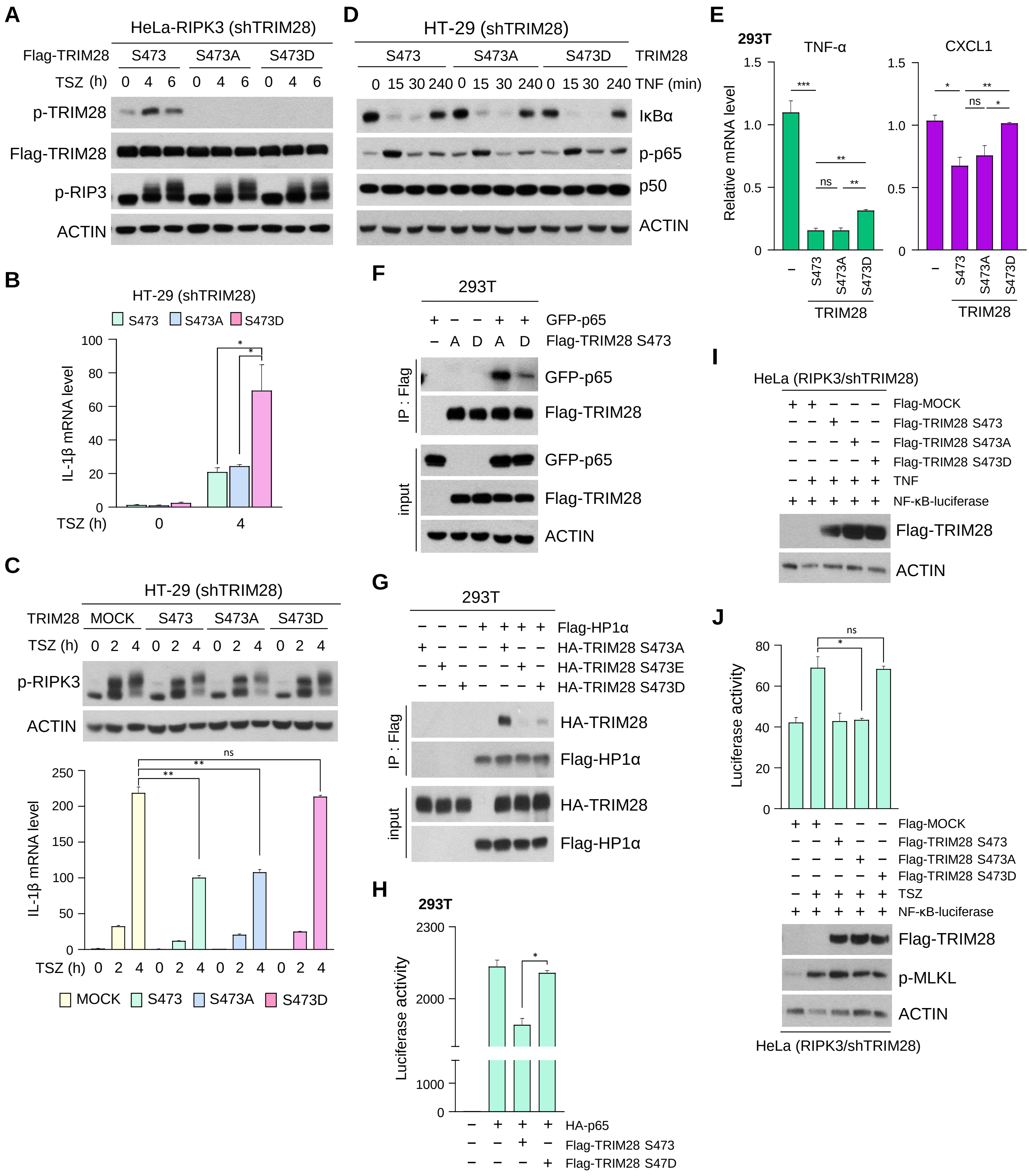

Supplementary Figure 5.

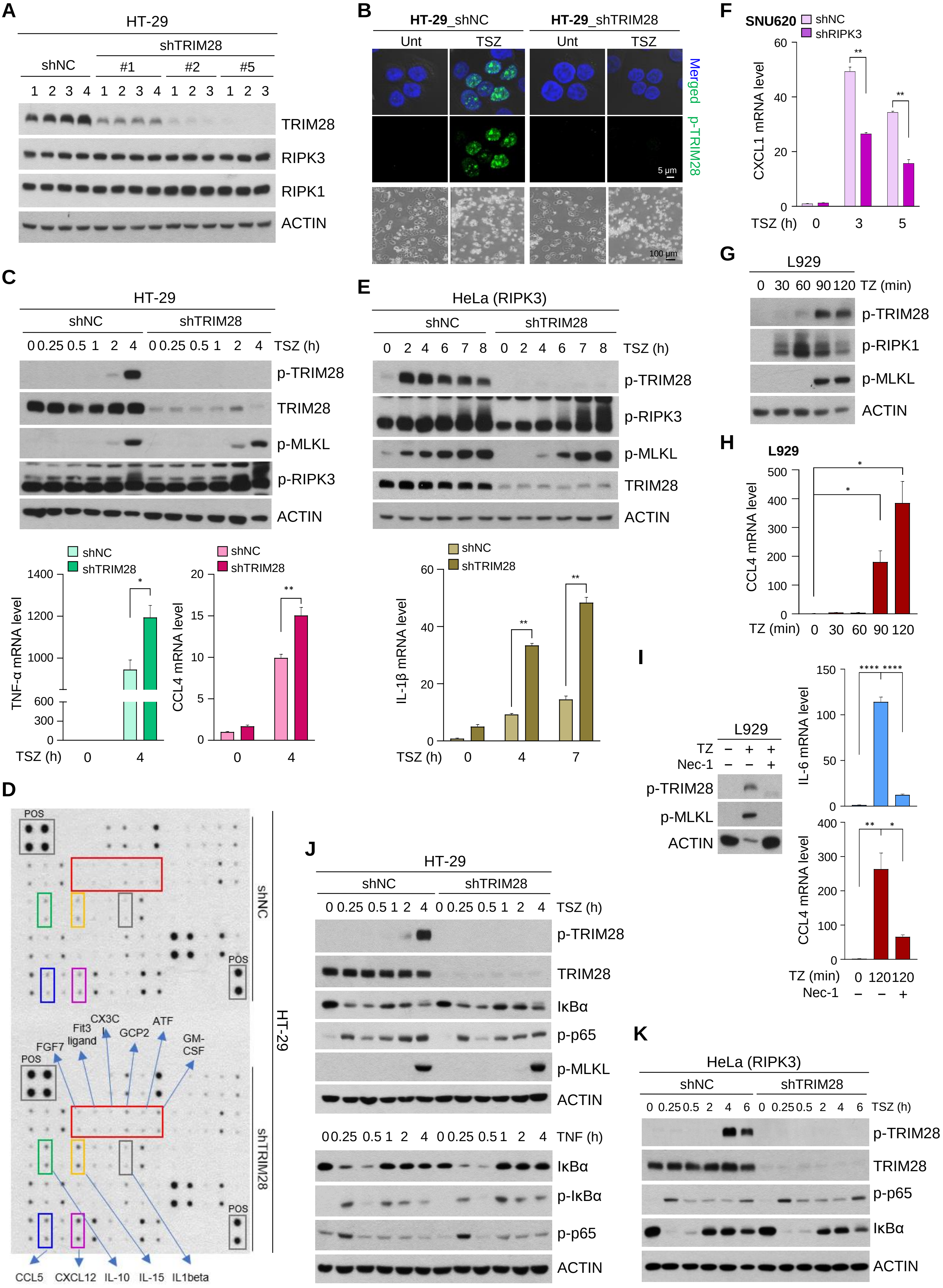

Supplementary Figure 6.

A

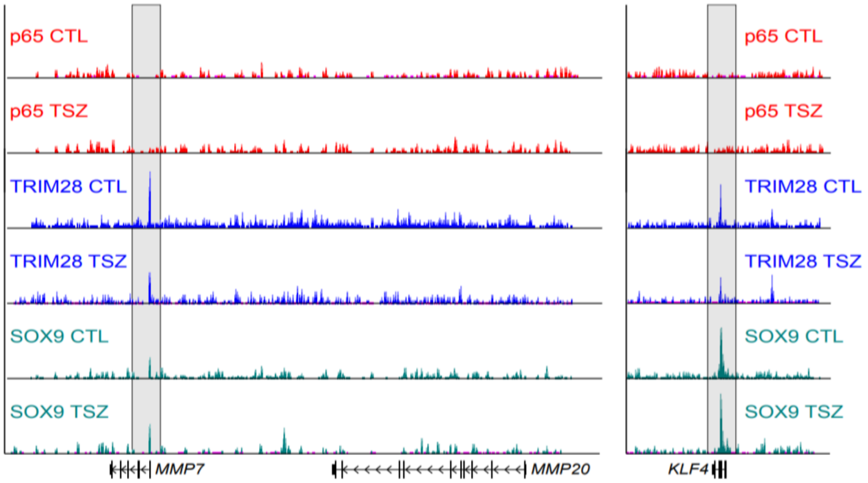

B

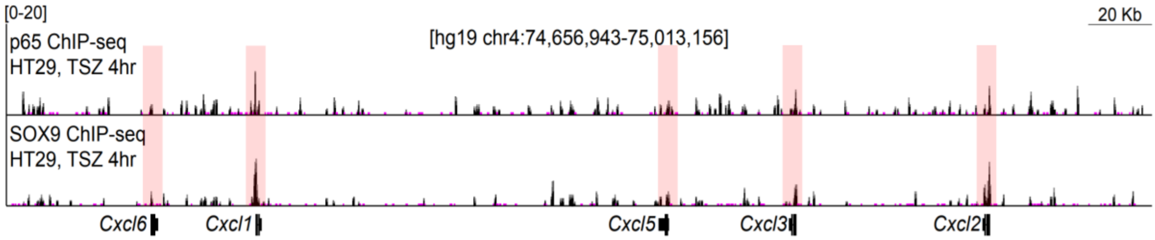

Supplementary Figure 7.

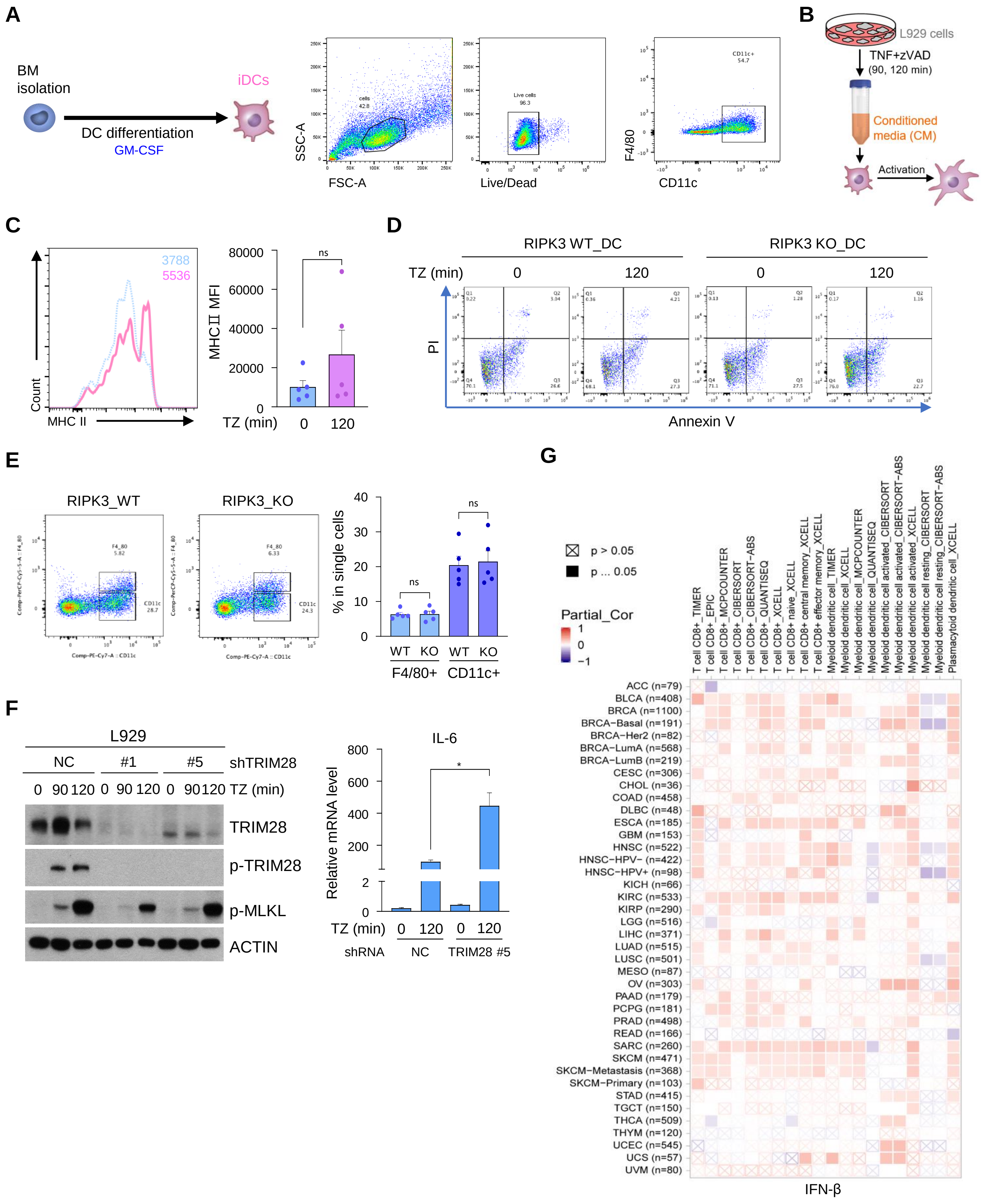

Supplementary Figure 8.

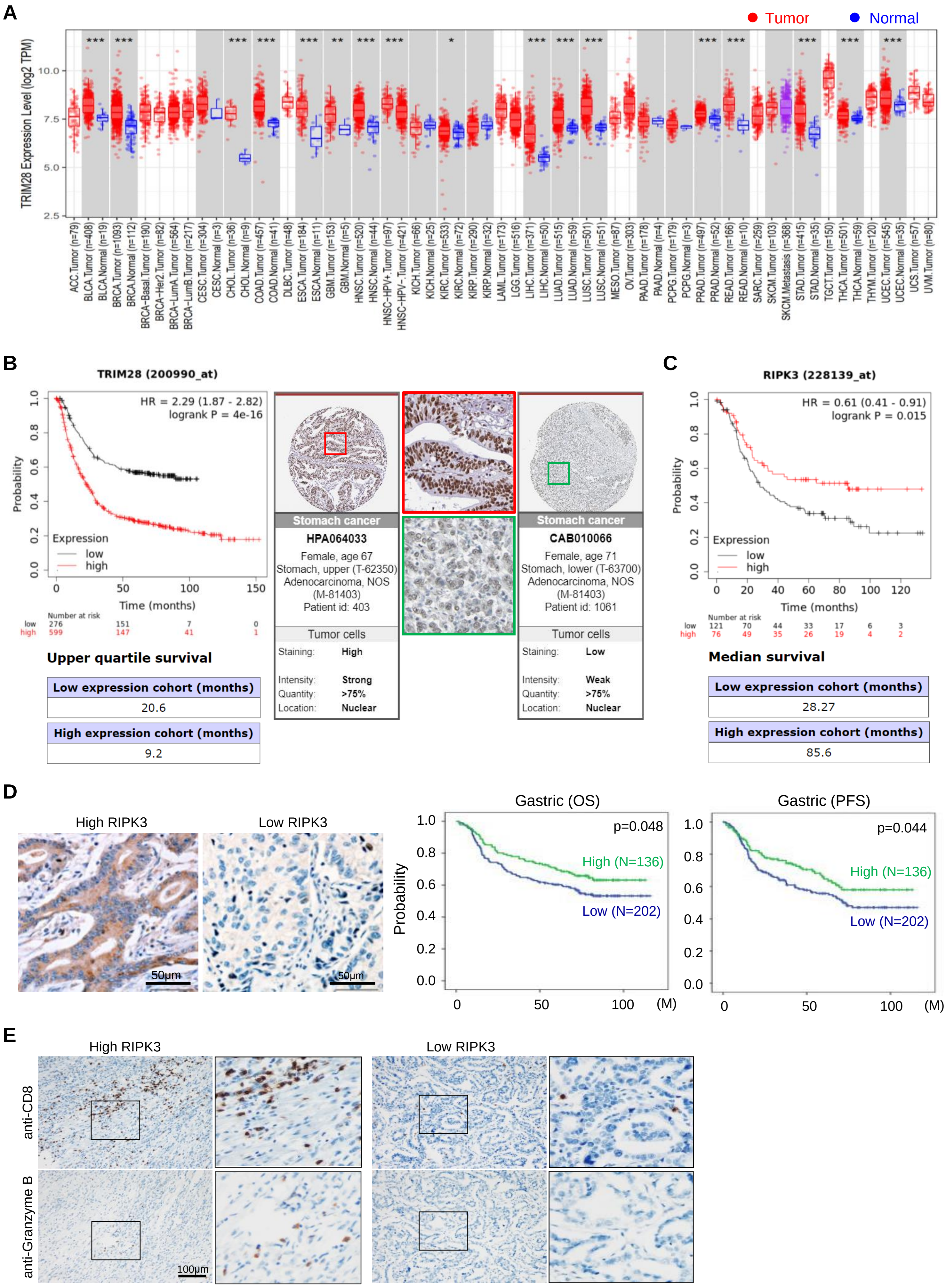

SUPPLEMENTAL INFORMATION

FIGURE LEGENDS

Figure S1. NF-κB transactivation in TNF- and TSZ-mediated downstream signals.

(A) Representation of motifs enriched at 163 genes associated with promoters. Known motif analysis was performed using HOMER; the top 5 ranked motifs are shown with their *p*-values.

(B) ChIP-seq profiles of p65 in HT-29 cells treated with TNF-α or TSZ for 30 min or 4 h at the *CXCL2, TNFAIP3, CSF1,* and *IL32* loci. HT-29 cells were treated with with TNF-α (30 ng/ml) or TSZ (TNF-α (30 ng/ml) + SMAC (200 nM) + zVAD (20 μM), hereafter referred to as TSZ.

(C) HT-29 cells were fractionated into nuclear and cytosol fractions after the indicated treatments and analyzed by western blotting.

(D) RNA-seq-based expression of Nec1-dependent 50 genes in HT-29 cells treated with TNF-α or TSZ.

(E) Scatter plot comparing the expression of the identified 50 genes in (D).

(F) Heatmap showing the expression of identified 50 genes in HT-29 cells treated with DMSO, TNF-α, TSZ, or Nec-1+TSZ.

(**G)** Metagene representation of the ChIP-seq signal for p65 across 164 regions, TNF-α & TSZ inducible/Nec1-dependent 50 genes-associated peaks in HT-29 cells treated with TNF-α or TSZ for 4 h. Metagenes centered on the middle of 164 regions and 10 Kb around the center of 164 regions are displayed (upper). Representation of motifs enriched at promoters associated with the identified 50 genes. The top 5 ranked motifs are shown with their *p*-values (bottom).

Figure S2. Interactome of active RIPK3.

(A) Visualization of gene list obtained from bioinformation analysis in Figure 2A. Data clock chart was generated by ArcGIS insight online program as indicated.

(B) 293T cells were co-transfected with GFP-TRIM28 and TAP-RIPK3. After 24 h, cell lysates were immunoprecipitated with GFP antibody.

(C) 293T cells were co-transfected with GFP-TRIM28 and TAP-RIPK3 WT or TAP-RIPK3 K50A for 24 h. Cell lysates immunoprecipitated with GFP antibody.

(D) 293T cells were co-transfected with Flag-RIPK3 and Myc-TRIM28 in the absence or presence of dabrafenib (DAB, 10 μM) for 24 h. Cell lysates were immunoprecipitated with Flag antibody.

Figure S3. RIPK3 activation-dependent phosphorylation of TRIM28 at serine 473.

(A) HT-29 cells were treated with etoposide or doxorubicin in a dose- (upper) and time-dependent manner (bottom). Cell lysates were analyzed by western blotting.

(B) HT-29 cells were treated with etoposide, doxorubicin, and TSZ for 4 h. Cell lysates were analyzed by western blotting.

(C) HeLa (RIPK3) cells were treated with TSZ for indicated time point and cell lysates were analyzed by western blotting.

(D) HT-29 cells were treated with TSZ for 4 h, immuno-stained, and visualized by confocal fluorescence microscopy (red: S473 phospho-TRIM28; blue: DAPI).

(E) TSZ-treated HeLa (RIPK3) cells were stained with S824 and S473 phospho-TRIM28

antibodies and analyzed by confocal fluorescence microscopy (green: S824 or S473 phospho-TRIM28; blue: DAPI).

(F) NIH-3T3 and RAW 264.7 cells were treated with TSZ for the indicated times. Cell lysates were analyzed by western blotting.

(G) HT-29, SNU620, and ML-1 cells were pretreated with Nec-1, GSK'872, and DAB for 1 h and with TSZ for the indicated times. Cell lysates analyzed by western blotting.

(H) MEF cells were pretreated Nec-1 and GSK'872 for 1 h and with TSZ for the indicated times. Cell lysates were analyzed by western blotting.

(I) HT-29 cells expressing RIPK3 shRNA or a non-silencing control were treated with TSZ for the indicated times. Cell lysates were analyzed by western blotting.

(J) ML-1 cells expressing RIPK3 shRNA or a non-silencing control were pretreated with Nec-1 and DAB for 1 h and treated with TSZ for 3 h. Cell lysates were analyzed by western blotting.

(K) RIPK3 wild-type and RIPK3-knockout MEFs were treated with TSZ for the indicated times. Cell lysates were analyzed by western blotting.

(L) HeLa cells stably expressing pLX-RIPK3 or the control vector (pLX-NC) were treated with TSZ for the indicated times. Cell lysates were analyzed by western blotting.

Figure S4. TRIM28 functions as a co-repressor in NF-κB transactivation.

(A) TRIM28-knockdown HeLa (RIPK3) cells were reconstituted with the TRIM28 S473 WT or TRIM28 S473 mutants and treated with TSZ for the indicated times. Cell lysates were analyzed by western blotting.

(B) TRIM28-knockdown HT-29 cells reconstituted with the TRIM28 S473 WT or TRIM28 S473 mutants were treated TSZ for the indicated times. *IL-1β* mRNA levels were measured by qPCR.

(C) TRIM28-knockdown HT-29 cells reconstituted with the vector or TRIM28 S473 mutants were treated with TSZ for the indicated times. Cell lysates were analyzed by western blotting (upper), and the expression levels of *IL-1β* mRNA were measured by qPCR (bottom).

(D) TRIM28-knockdown HT-29 cells reconstituted with the TRIM28 S473 WT or TRIM28 S473 mutants were treated with TNF-α for the indicated times. Cell lysates analyzed by western blotting.

(E) 293T cells were transiently transfected with Flag-TRIM28 S473 WT or the S473 mutants. After 24 h, *TNF-α* and *CXCL1* mRNA levels were measured by qPCR.

(F) 293T cells were co-transfected with Flag-TRIM28 S473 mutants and GFP-p65. After 24 h, cell lysates were immunoprecipitated with Flag antibody.

(G) 293T cells were co-transfected with HA-TRIM28 S473 mutants and Flag-HP1α. After 24 h, cell lysates were immunoprecipitated with Flag antibody.

(H) 293T cells were transiently co-transfected with HA-p65 and Flag-TRIM28 S473 WT or the Flag-TRIM28 S473D mutant. After 24 h, luciferase assays were performed.

(I) TRIM28-knockdown HeLa (RIPK3) cells were transiently transfected with Flag-TRIM28 S473 WT and S473 mutants. After 24 h of transfection, cells were treated with TNF-α for 6 h. Cell lysates were analyzed by western blotting.

(J) TRIM28-knockdown HeLa (RIPK3) cells were transiently transfected with Flag-TRIM28 S473 WT and S473 mutants. After 24 h, cells were treated with TSZ for 6 h. Luciferase activity was measured.

Figure S5. TRIM28 negatively regulates necrosome-induced cytokine production.

(A) HT-29 cells expressing different sequences (#1, #2, #5) of TRIM28 shRNA or a non-silencing control. were analyzed by western blotting.

(B) HT-29 cells expressing TRIM28 shRNA (#5) or a non-silencing control were treated with TSZ for 4 h, stained with S473 phospho-TRIM28 antibody, and visualized by confocal fluorescence microscopy (green: S473 phospho-TRIM28; blue: DAPI).

(C) HT-29 cells expressing TRIM28 shRNA or a non-silencing control were treated with TSZ for the indicated times. Cell lysates were analyzed by western blotting (upper) and *TNF-α* and *CCL4* mRNA levels were measured by qPCR (bottom).

(D) Cytokine array showed upregulated cytokine production in TRIM28 knock-downed cells. HT-29 cells expressing TRIM28 shRNA or a non-silencing control were treated with TSZ for 6 h and supernatant were used on each array blot. The duration of exposure was compared with positive control (POS). Color square boxes indicate the increased signals in supernatant from shTRIM28 cells than non-silencing control cells.

(E) TRIM28-knockdown HeLa (RIPK3) cells were treated with TSZ for the indicated times. Cell lysates were analyzed by western blotting (upper) and *IL-1β* mRNA levels were measured by qPCR (bottom).

(F) SNU-620 cells expressing RIPK3 shRNA or a non-silencing control were treated with TSZ for the indicated times. *CXCL1* mRNA levels were measured by qPCR.

(G and H) L929 cells were treated with TZ for the indicated times, and cell lysates analyzed by

western blotting (G) and *CCL4* mRNA levels were analyzed by qPCR (H).

(I) L929 cells were pretreated with Nec-1 (40 μM) for 1 h and treated with TZ for 2 h. Cell lysates were analyzed by western blotting (left), and *IL-6* and *CCL4* mRNA levels were analyzed by qPCR (right).

(J) HT-29 cells expressing TRIM28 shRNA or a non-silencing control were treated with TSZ (upper) or TNF-α (bottom) for the indicated time points. Cell lysates were analyzed by western blotting.

(K) HeLa (RIPK3) cells expressing TRIM28 shRNA or a non-silencing control were treated with TSZ for the indicated time points and cell lysates were analyzed by western blotting.

Figure S6. SOX9 is a potential transcription factor of necroptosis-induced cytokine production.

(A and B) ChIP-seq profiles of p65, TRIM28, and SOX9 in HT-29 cells treated with DMSO or TSZ for 4 h at the *MMP7* and *KLF4* loci (A), or *CXCL* gene cluster (B).

Figure S7. Increased cytokine production by RIPK1/RIPK3 activation contributes to DC activation.

(A) Schematic representation of DC differentiation (left). Gating strategies for FACS plots (right).

(B) Preparation of the conditioned medium (CM) for iDC activation.

(C) DCs were treated with the CM for 16 h and then MHC II was analyzed by flow cytometry.

(D) DCs from RIPK3 WT or RIPK3 KO mice were treated with the CM for 16 h, stained with PI/Annexin V, and subjected to FACS analysis.

(E) CD11c, and F4/80 events were quantified by FACS in DCs from RIPK3-WT or RIPK3-KO mice.

(F) L929 cells stably expressing TRIM28 shRNA or a non-silencing control were treated with TZ for the indicated times. Cell lysates were analyzed by western blotting (left), and *IL-6* mRNA levels were analyzed by qPCR (right).

(G) The heatmap table shows the association between IFN-β and tumor-infiltrating level of multiple types of $CD8^+$ T cell or DCs, and that is estimated by six algorithms across cancer types in the TCGA database. Red indicates a statistically significant positive association, and blue indicates a statistically significant negative association.

Figure S8. RIPK3/TRIM28 may be a potent target for necroptosis-mediated anti-tumor immunity.

(A) TRIM28 expression level between tumor and adjacent normal tissues across all TCGA tumors. The statistical significance computed by the Wilcoxon test (*: p-value < 0.05; **: p-value <0.01; ***: p-value <0.001).

(B) TRIM28 expression correlates with clinical outcome in gastric cancer patients. Kaplan-Meier curve showing the overall survival (OS) of gastric cancer patients possessing *TRIM28* mRNA expression either above or below the median (left). Protein expression of TRIM28 in tissues from gastric cancer patients (Modified from Human Atlas, right).

(C) RIPK3 expression correlates with clinical outcome in stage 3 gastric cancer patients. Kaplan-Meier curve showing the overall survival (OS) of stage 3 gastric cancer patients possessing *RIPK3* mRNA expression either above or below the median.

(D) RIPK3 expression is correlated with clinical outcome in gastric cancer patients. Representative photomicrographs of immunohistochemical staining for RIPK3 in paraffin-embedded tumor samples from gastric cancer patients, who underwent curative surgery in Ajou University Hospital (left). Kaplan-Meier analyses exhibit overall survival (OS) and progression-free survival (PFS) according to the high or low RIPK3 expression (N=338). OS time was defined as the interval between the date of surgery and death. PFS time was defined as the interval between the date of surgery and the first recurrence or death (right).

(E) Tissue samples with high RIPK3 expression show strong reactivity to anti-CD8 and anti-Granzyme B antibodies in the same cohort (D).
